# Tensin3 interaction with talin drives the formation of fibronectin-associated fibrillar adhesions

**DOI:** 10.1083/jcb.202107022

**Published:** 2022-09-08

**Authors:** Paul Atherton, Rafaella Konstantinou, Suat Peng Neo, Emily Wang, Eleonora Balloi, Marina Ptushkina, Hayley Bennett, Kath Clark, Jayantha Gunaratne, David Critchley, Igor Barsukov, Edward Manser, Christoph Ballestrem

**Affiliations:** 1 Wellcome Trust Centre for Cell-Matrix Research, University of Manchester, Manchester, UK; 2 Division of Cell Biology, The Netherlands Cancer Institute, Amsterdam, The Netherlands; 3 sGSK Group, Institute of Molecular and Cell Biology, A*STAR (Agency for Science, Technology and Research), Singapore, Singapore; 4 Quantitative Proteomics Group, Institute of Molecular and Cell Biology, A*STAR (Agency for Science, Technology and Research), Singapore, Singapore; 5 Institute of Integrative Biology, University of Liverpool, Liverpool, UK; 6 Genome Editing Unit, Faculty of Biology, Medicine and Health, University of Manchester, Manchester, UK; 7 Department of Biochemistry, University of Leicester, Leicester, UK

## Abstract

The formation of healthy tissue involves continuous remodeling of the extracellular matrix (ECM). Whilst it is known that this requires integrin-associated cell-ECM adhesion sites (CMAs) and actomyosin-mediated forces, the underlying mechanisms remain unclear. Here, we examine how tensin3 contributes to the formation of fibrillar adhesions (FBs) and fibronectin fibrillogenesis. Using BioID mass spectrometry and a mitochondrial targeting assay, we establish that tensin3 associates with the mechanosensors such as talin and vinculin. We show that the talin R11 rod domain binds directly to a helical motif within the central intrinsically disordered region (IDR) of tensin3, whilst vinculin binds indirectly to tensin3 via talin. Using CRISPR knock-out cells in combination with defined tensin3 mutations, we show (i) that tensin3 is critical for the formation of α5β1-integrin FBs and for fibronectin fibrillogenesis, and (ii) the talin/tensin3 interaction drives this process, with vinculin acting to potentiate it.

## Teaser

Atherton and Konstantinou et al. reveal the molecular mechanisms underpinning an interaction between the integrin-associated proteins tensin3 and talin. By associating with the intracellular force-generating actomyosin machinery, this talin–tensin3 interaction drives the formation of fibrillar adhesions and associated fibronectin fibrils as part of the extracellular matrix remodeling.

## Introduction

Cells interact with the extracellular matrix (ECM) through transmembrane integrin receptors that connect with the actin cytoskeleton through multi-protein complexes. Integrin-mediated cell-matrix adhesions (CMAs) can vary in location, shape, and function. CMAs initially form at the cell periphery as dot-like focal complexes that develop into focal adhesions (FAs) through actomyosin-mediated tension. FAs can mature further into centrally located fibrillar adhesions (FBs). In contrast to the well-established roles of FAs in sensing both biophysical and biochemical ECM cues, rather little is known about the formation and function of FBs.

FBs are intimately linked to fibronectin fibrils and other associated ECM proteins, such as collagens, the organization of which depends on integrins (Clark et al., 2005; Green et al., 2009; Lu et al., 2020; Pankov et al., 2000). The formation of fibronectin fibrils has been observed in both fibroblasts and epithelial cells (Lu et al., 2020), and involves the translocation of fibronectin-bound α5β1 integrins from peripheral FAs to central FBs (Clark et al., 2005; Pankov et al., 2000) in a tension-dependent manner (Lu et al., 2020; Zamir et al., 2000). The force-mediated maturation of FAs to FBs is accompanied by a switch in molecular composition: FAs are enriched in talin, vinculin, paxillin, and FAK; FBs predominantly contain α5β1 integrins and tensin1 and 3 (Clark et al., 2010; Zamir et al., 2000). While overexpression of a dominant negative chicken tensin fragment and tensin depletion experiments show that tensins are important for fibronectin fibrillogenesis (Georgiadou et al., 2017; Pankov et al., 2000), mechanistic insights are lacking.

**Figure S1.**
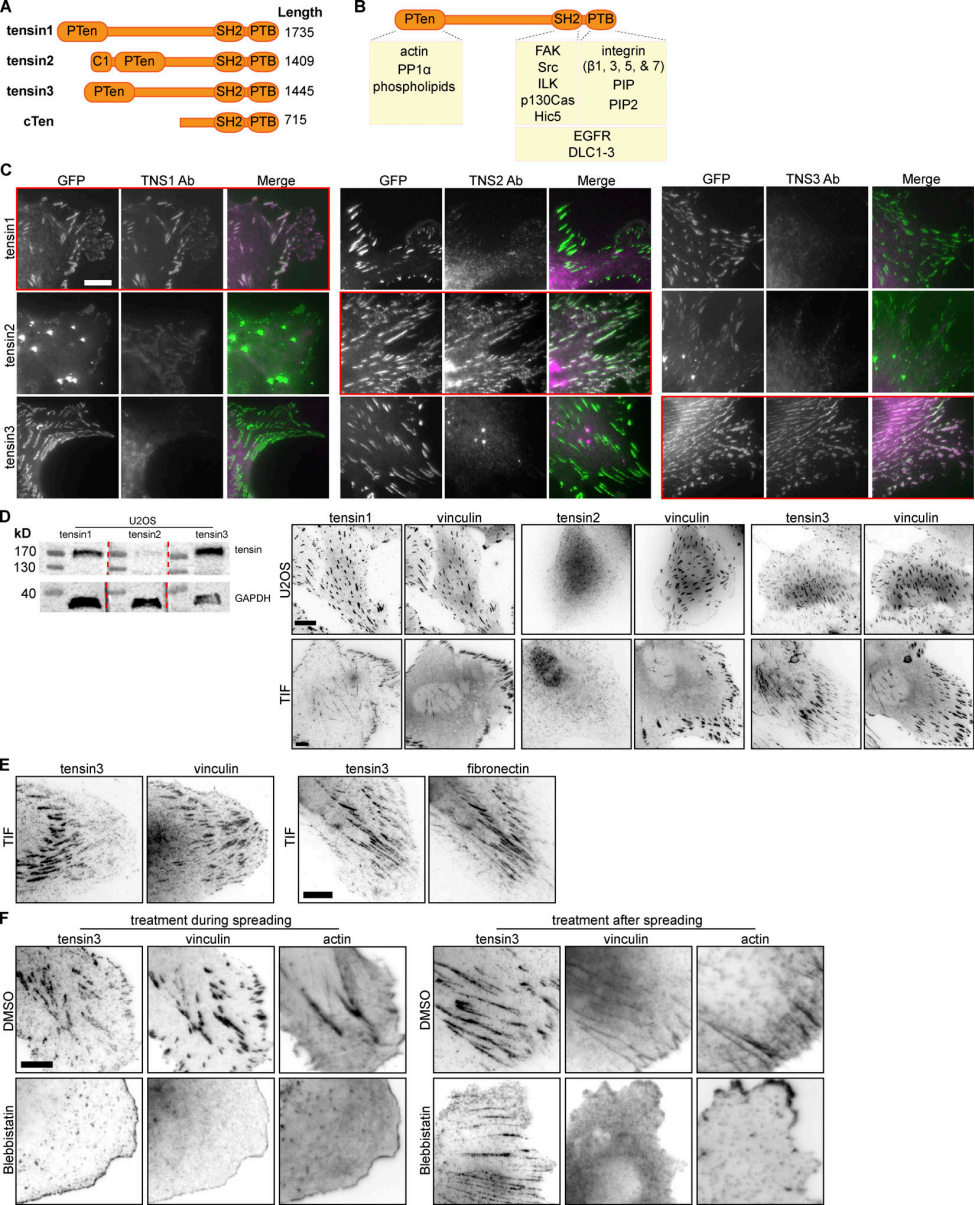
**Characterisation of tensin specific antibodies and tensin localization. (A)** Schematic of the four human tensin family members. Tensin1-3 have an N-terminal region homologous to phosphatase and tensin homolog (PTEN). Tensin2 has an N-terminal lipid-binding C1 domain. All four family members have C-terminal Src-homology 2 (SH2) and phosphotyrosine-binding (PTB) domains. The middle regions are largely unstructured with little amino acid sequence homology between family members. **(B)** Schematic showing various reported interaction partners of the tensin family, including: cytoplasmic domains of beta integrin subunits (β1, β3, β5, β7) with the PTB domain that reportedly supports integrin activation; the SH2 domain with p130CAS; actin to the N-terminus; the Rho GAPs DLC1-3 to both the N- and C-termini; growth factor receptors (EGFR, cMET) and additional FA proteins (e.g., FAK and p130Cas, Hic5 and ILK) to the C-terminus. **(C)** NIH3T3 mouse fibroblasts were transfected with the indicated GFP-tagged human tensin constructs and immunostained with antibodies against (human) TNS1, TNS2, or TNS3. Note that the respective antibodies detect only the expressed tensin member they are directed against (i.e., tensin1 antibody detects only over-expressed GFP-tensin1, tensin2 antibody only the expressed GFP-tensin2 and the tensin3 antibody only the GFP-tensin3; indicated by red boxes). **(D)** Expression of tensin1, 2, and 3 in U2OS and TIF cells evaluated by Western blot and immunofluorescence. A whole cell lysate from U2OS cells was run on the same gel separated by ladder lanes. After transfer, the membrane was cut and incubated with individual tensin antibodies and GAPDH separately (since tensin1, 2, and 3 have a similar molecular weight). **(E)** Left panel: TIF cell stained for vinculin and tensin3, note that tensin3 localises more to the centrally located adhesions and vinculin to the peripheral adhesions; right panel: TIF cell stained for tensin3 and fibronectin, note the similar localization. **(F)** Left panels: TIF cells were treated in suspension with blebbistatin (50 µM) or an equivalent volume of DMSO, for 60 min. Cells were fixed after spreading on fibronectin-coated glass for 60 min. Note the absence of tensin3- or vinculin-positive structures in blebbistatin-treated cells. Right panels: TIF cells cultured overnight on fibronectin-coated glass were treated with blebbistatin (50 µM) or an equivalent volume of DMSO, for 45 min prior to fixation. Scale bars in C–F indicate 10 µm. Source data are available for this figure: SourceData FS1.

In contrast to the single tensin gene in chickens, mammals have four tensin genes (tensin1-4), three of which (tensin1-3) encode structurally similar proteins (Fig. S1 A). Expression of fluorophore-tagged human tensin1-3 in fibroblasts revealed variations in localization to different CMA types. Whilst tensin2 predominantly localized to FAs and tensin3 to FBs, tensin1 was in both structures (Clark et al., 2010). What regulates this difference remains unclear given their structural similarities. Although several proteins have been found to bind to the highly conserved N- and C- termini of the tensins (Calderwood et al., 2003; Cao et al., 2012; Cui et al., 2004; Goreczny et al., 2018; Hall et al., 2010; Katz et al., 2007; Kawai et al., 2009; Liao and Lo, 2021; Liao et al., 2007; Lo et al., 1994; McCleverty et al., 2007; Muharram et al., 2014; Qian et al., 2007; Shih et al., 2015; Zhao et al., 2016; Fig. S1 B), most of the interaction studies involved binding to isolated peptides, with little evidence that the interactions occur in cells or are linked to specific biological functions.

In this study, we aimed to gain a mechanistic understanding of the role of tensins in FB formation and its contribution to fibronectin fibrillogenesis. We focussed on tensin3, the family member with the strongest enrichment in FBs (Clark et al., 2010). Using a combination of proximity biotinylation mass spectrometry (BioID) and fluorescence microscopy, we identified several potential tensin interactors including the mechanosensors talin and vinculin. In vitro nuclear magnetic resonance (NMR) and isothermal titration calorimetry (ITC) experiments showed that the intrinsically disordered region (IDR) of tensin3 contains a talin binding motif similar to those previously identified in DLC1 and RIAM (Goult et al., 2013). Complementary cell biological assays showed that the tensin3–talin interaction is critical for the force-mediated maturation of FBs and fibronectin fibrillogenesis, processes potentiated by vinculin binding to talin.

## Results

### Actomyosin-mediated forces are required for the formation of FBs but not their maintenance

**Figure 1.**
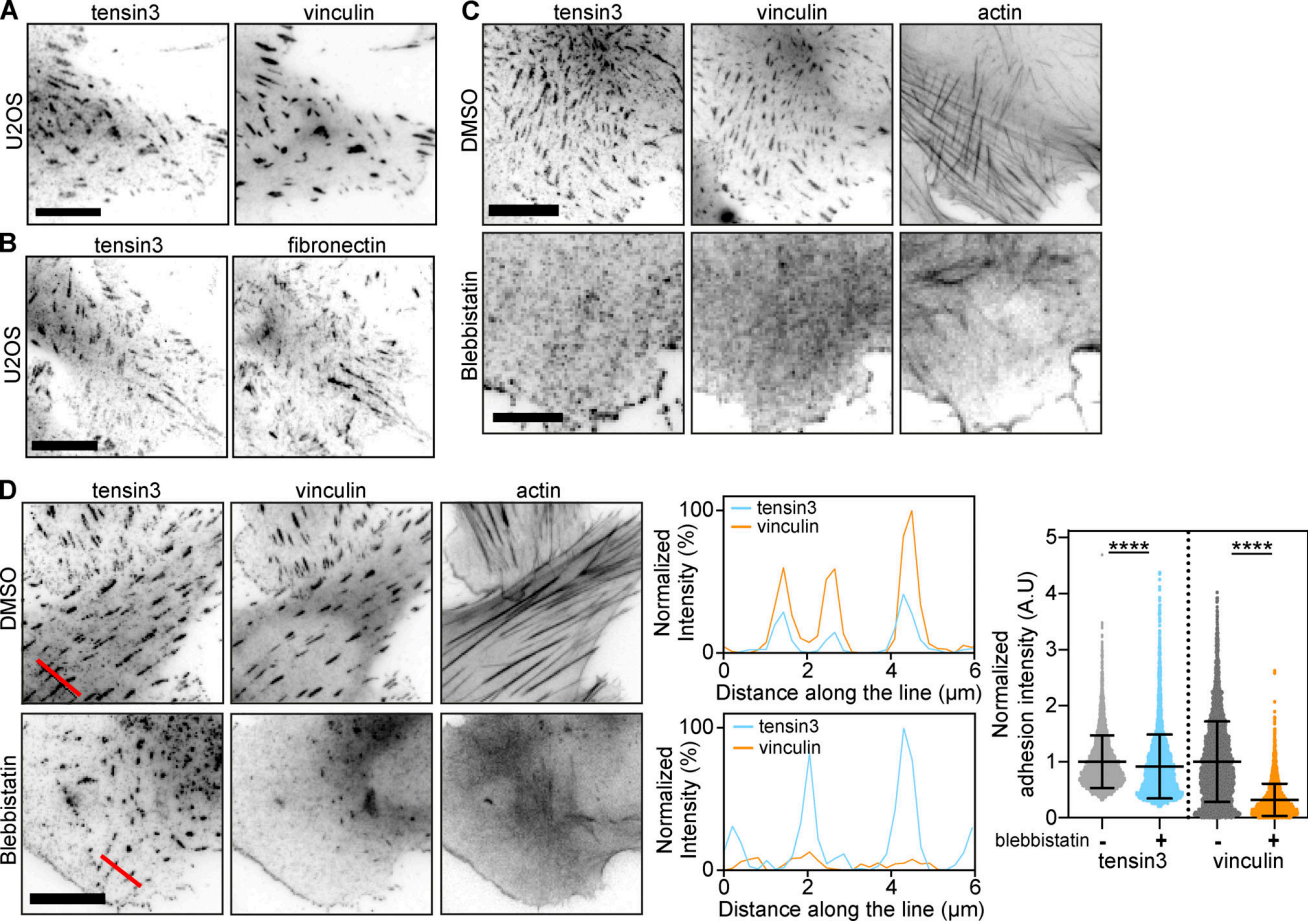
**Actomyosin-mediated forces are required for the formation of tensin3-positive fibrillar adhesions. (A and B)** Representative immunofluorescence images of U2OS cells cultured overnight on fibronectin-coated glass stained for (A) tensin3 and vinculin, or (B) tensin3 and fibronectin. **(C)** U2OS cells were treated in suspension with blebbistatin (50 µM) or an equivalent volume of DMSO, for 60 min. Cells were fixed after spreading on fibronectin-coated glass for 4 h. Note the absence of tensin3- or vinculin-positive structures in blebbistatin-treated cells. **(D)** U2OS cells cultured overnight on fibronectin-coated glass were treated with blebbistatin (50 µM) or an equivalent volume of DMSO, for 60 min before fixation. Line profiles indicate fluorescences intensity levels of proteins from background subtracted FA images. Note that tensin3 remains at adhesions whilst vinculin disappears after blebbistatin treatment. Data are normalized to control (DMSO-treated) conditions individually for tensin and vinculin. Error bars are SD; *n* = 5,206 (DMSO); 4,078 (Blebbistatin) adhesions from 45 to 56 cells respectively; data were pooled from three independent experiments; **** indicates P < 0.0001 (Mann Whitney *t* test). Scale bars in A–C indicate 10 µm.

Immunostaining of U2OS osteosarcoma cells or telomerase-immortalized fibroblasts (TIFs) with antibodies specific for the different tensin isoforms (for the characterization of antibody specificity see Fig. S1 C) revealed that both cell types expressed tensin1 and tensin3, but little tensin2 (Fig. S1 D). As reported for other cell types (Clark et al., 2010), tensin3 was enriched at centrally located CMAs, co-aligning with fibronectin fibrils, whereas vinculin was more prominent in peripheral adhesions (Fig. 1, A and B; and Fig. S1 E).

FBs were previously shown to develop from FAs via myosinII-dependent gliding of α5β1 integrins (Lu et al., 2020). We confirmed that a similar mechanism regulates tensin3 localization by treating U2OS cells (Fig. 1 C ) or TIFs (Fig. S1 F) with the actomyosin inhibitor blebbistatin (50 µM) before the cells spread on fibronectin. Under these conditions, neither vinculin-positive FAs nor tensin3-enriched FBs formed (Fig. 1 C). In contrast, treatment of U2OS cells that had already spread on fibronectin (and formed tensin3-positive FBs) with blebbistatin maintained a large number of tensin3-positive structures with many of the vinculin-positive FAs disappearing (8% reduction in tensin compared to 68% reduction in vinculin compared to their respective DMSO samples, Fig. 1 D); similar results were seen for TIF cells (Fig. S1 F). Interestingly, the stoichiometry of tensin versus vinculin in these remaining adhesions changed dramatically showing that vinculin leaves adhesions whereas a large fraction of tensin remains residual in these adhesions (line profiles and quantification in Fig. 1 D). The data demonstrate that the formation of FBs depends on forces, but their maintenance is largely independent of intracellular tension.

### Identification of tensin binding partners

**Figure S2.**
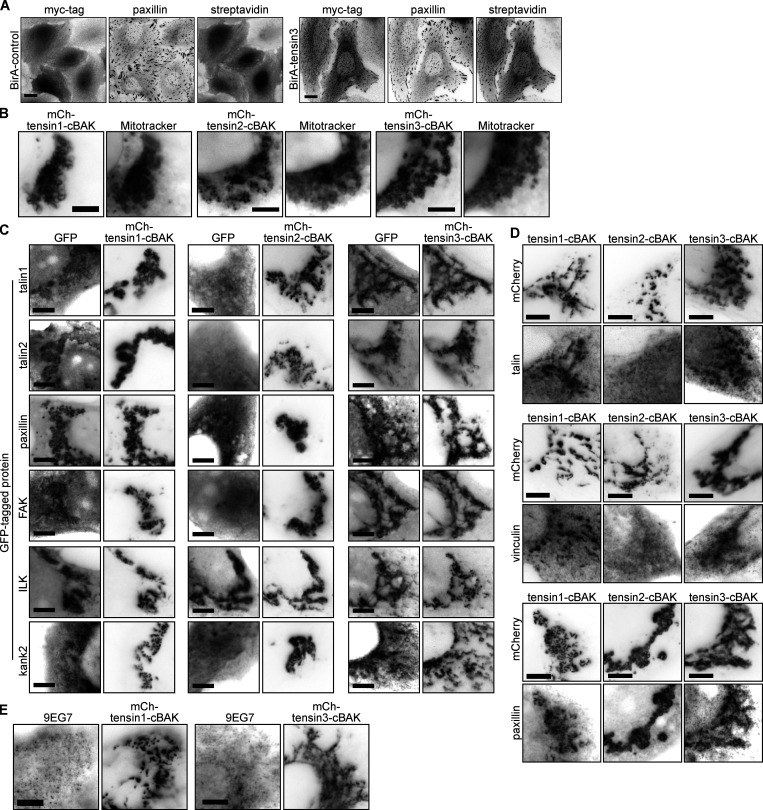
**The mitochondrial targeting system confirms tensin interactions with other FA proteins. (A)** U2OS cells stably expressing BirA-control or BirA-tensin3 were incubated with biotin for 16 h before being fixed and stained for myc and paxillin and biotinylated proteins (using fluorophore [Dylight 488]-conjugated streptavidin). Scale bar indicates 10 µm. **(B)** The C-terminus of tensin1, 2, or 3 was fused to the short mitochondrial targeting sequence from the outer mitochondrial membrane protein BAK (cBAK), with an N-terminal mCherry (mCh) tag. When expressed in NIH3T3 cells each of these constructs co-localize with the mitochondria-specific dye MitoTracker. **(C)** Representative images of NIH3T3 cells co-expressing the indicated GFP-tagged cell-matrix adhesion protein together with the indicated mCh-tensin-cBAK construct. **(D)** Representative images of NIH3T3 cells expressing mCh-tensin-cBAK constructs immunostained for endogenous talin, vinculin or paxillin. **(E)** Representative images of NIH3T3 cells expressing mCh-tensin1-cBAK or mCh-tensin3-cBAK immunostained for active β1 integrin (9EG7 stain) reveals the absence of active integrins. Scale bars in B–E indicate 5 µm.

To better understand the mechanisms underlying the formation of tensin3-enriched central adhesions, we examined the molecular neighborhood of tensin3 using SILAC (stable isotope labeling by amino acids) BioID which labels proteins within a 1–20 nm radius of the bait protein (Dong et al., 2016). We stably expressed tensin3 with an N-terminal BirA* plus myc tag in U2OS cells (BirA-tensin3); cells expressing myc-BirA* alone (BirA-control) were used as controls. Immunofluorescence showed that BirA-tensin3 localized prominently in FBs, and a similar pattern was seen for biotinylation (Fig. S2 A), indicating the construct is functional.

**Figure 2.**
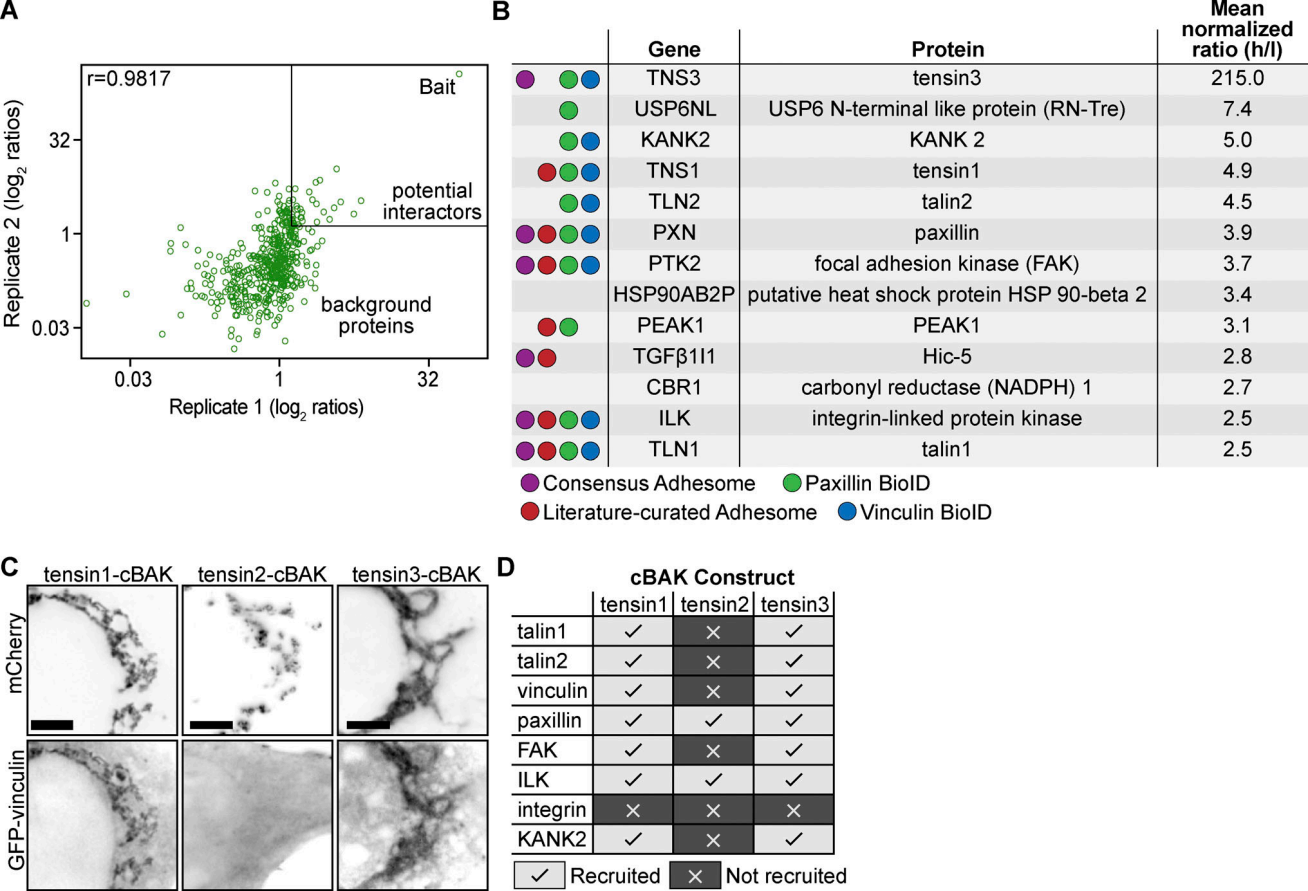
**Tensin3 interaction partners revealed by proximity biotinylation (A and B) and the mitochondrial targeting system (C and D). (A)** Scatter plot showing the correlation of the SILAC colocalization log_2_ ratio of the two tensin3 BioID repeats (data set in file Table S1). The majority of proteins are background proteins (bottom left of the graph); those on the top right of the graph (SILAC ratio ≥2.5) were considered as enriched. The two repeats were highly reproducible with a correlation coefficient of *r* = 0.9817. **(B)** Table listing the proteins identified from the BioID experiments using a cut-off ≥2.5 (Ratio of Heavy/Light). **(C)** Co-expression of mCh-tensin1-, tensin2-, or tensin3-cBAK with GFP-vinculin in NIH3T3 cells shows that GFP-vinculin co-localizes at mitochondria with both tensin1- and tensin3-cBAK but not tensin2-cBAK. **(D)** Summary table of which cell-matrix adhesion proteins co-localised with mCh-tensin1-, tensin2-, or tensin3-cBAK in NIH3T3 cells; scale bar indicates 10 µm.

Mass spectrometry analysis using normalized heavy-to-light ratios as an index for molecular proximities with a threshold ≥2.5 identified 13 proteins in the 20 nm radius proximal to the N-terminal of tensin3 (Fig. 2 A). Comparison with literature curated (Zaidel-Bar et al., 2007) and experimental datasets (Chastney et al., 2020; Dong et al., 2016; Horton et al., 2015) demonstrated that the majority of these tensin “neighbors” are known bona fide CMA proteins (Fig. 2 B). Other FA proteins, such as vinculin, LPP, and kindlin-2, were found in a list of proteins with a heavy-to-light ratio threshold ≥1.0 (Table S1). Together, these data indicate that tensin3 is embedded in a dense network of CMA proteins.

To validate a number of these potential interactions and to extend our findings to tensin1 and 2, we performed experiments using a mitochondrial targeting and recruitment system (Atherton et al., 2020). For these assays, tensin1–3 were tagged with mCherry at their N-termini and with the cBAK mitochondrial targeting sequence (Atherton et al., 2020) at their C-termini. When expressed in NIH3T3, fibroblasts all of these tensin constructs localized to the mitochondria (Fig. S2 B). We then examined the ability of putative GFP-tagged tensin binding partners to colocalize with mCherry-tensin-cBAK at the mitochondria (Fig. 2 C and Fig. S2 C). Interestingly, both tensin1 and tensin3 recruited talin (talin1 and 2), vinculin, paxillin, FAK, KANK2, and ILK to the mitochondria, whereas tensin2 only recruited ILK and paxillin (Fig. 2 D). Immunofluoresence confirmed the co-localization of endogenous talin1, vinculin, and paxillin with tensin1 and 3 at the mitochondria (Fig. S2 D). Integrins (active β1; 9EG7 staining) were absent from the mitochondria (Fig. S2 E), indicating that the associations occur independently of the reported tensin-integrin association (Georgiadou et al., 2017; McCleverty et al., 2007).

### Talin and vinculin associate with the tensin3-IDR

**Figure 3.**
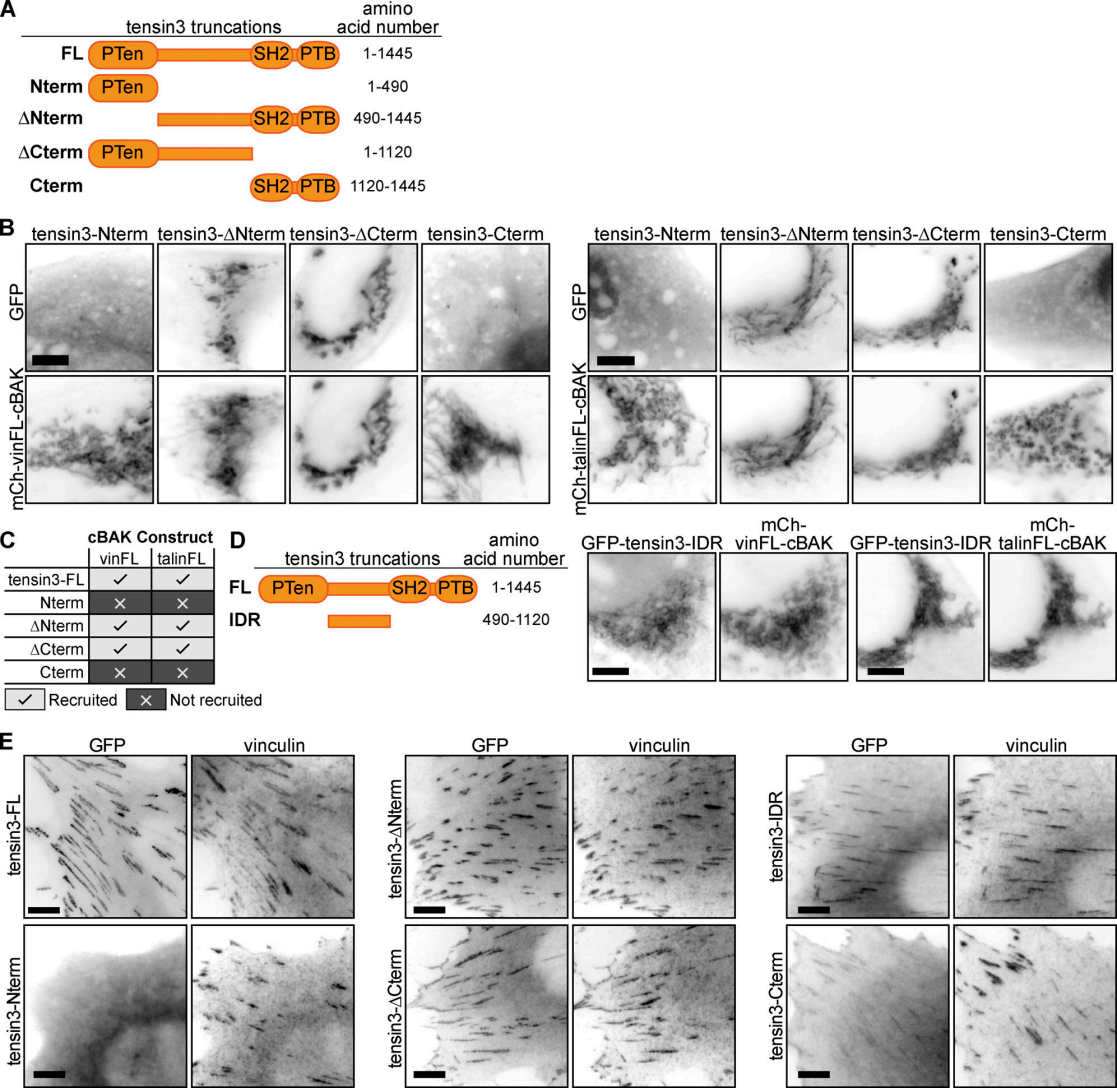
**The tensin3 IDR is required for the interaction with talin and vinculin. (A)** Schematic of the tensin3 deletion mutants used, with indicated truncation sites. **(B and C)** Tensin3 truncations were expressed as N-terminally tagged GFP fusion constructs together with mCh-vinFL-cBAK or mCh-talinFL-cBAK in NIH3T3 cells. Only those constructs containing the intrinsically disordered region (IDR) of tensin3 linking the PTEN and SH2 domains co-localized with vinculin or talin at mitochondria. **(D)** A tensin3 IDR-only construct (GFP-tensin3-IDR) co-localizes at mitochondria when co-expressed in NIH3T3 cells with either mCh-vinFL-cBAK or mCh-talinFL-cBAK. **(E)** GFP-tagged tensin3 truncations expressed in NIH3T3 cells; strongest localization to cell-matrix adhesions is observed in those constructs containing the tensin3 IDR (GFP-tensin3-∆Nterm, GFP-tensin3-∆Cterm, GFP-tensin3-IDR). Scale bars in B, D, and E indicate 10 µm.

**Figure S3.**
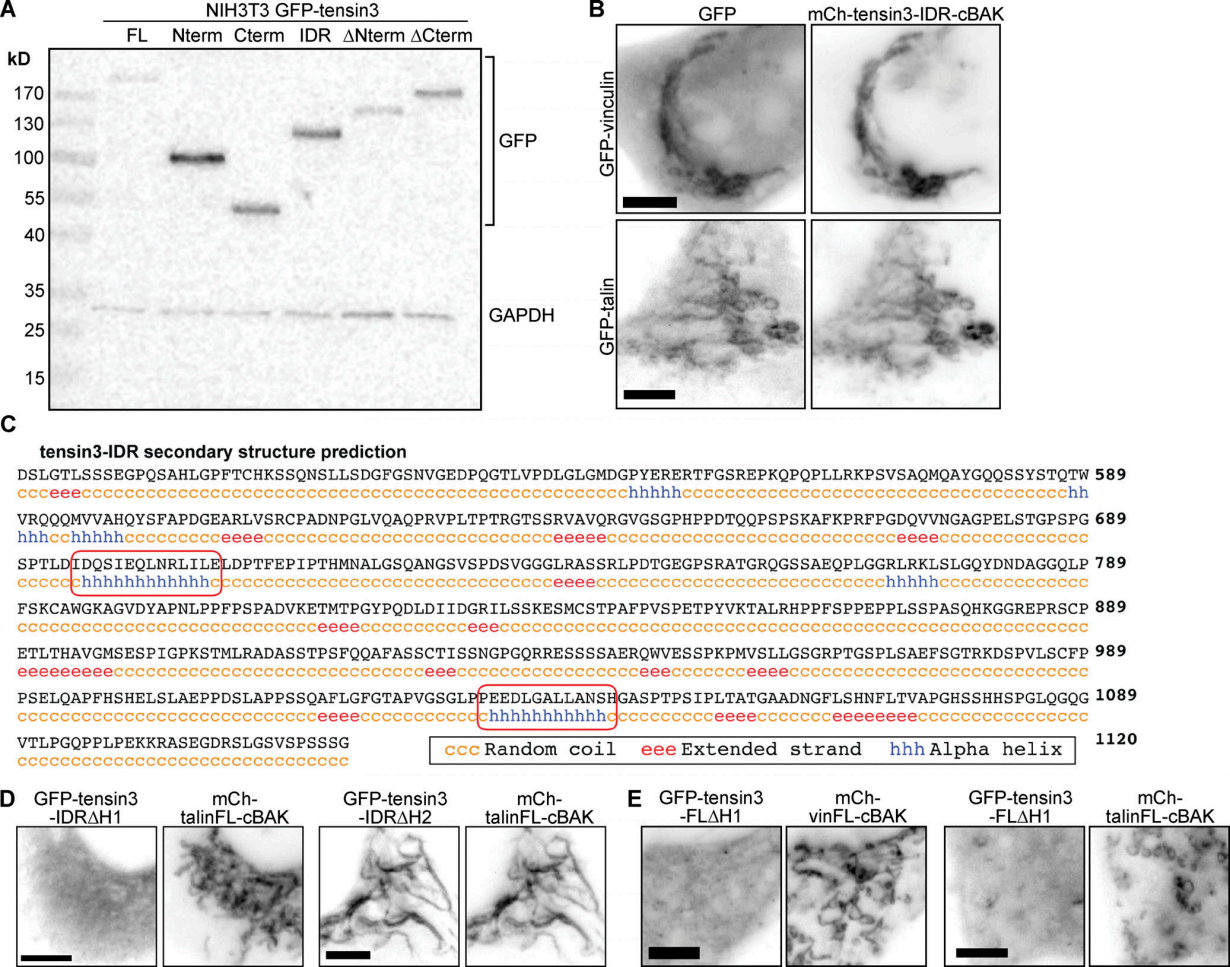
**Talin and vinculin interact with the IDR of tensin3. (A)** Western blot of tensin3 constructs N-terminally tagged with GFP expressed in NIH3T3 cells. **(B)** Co-expression of mCh-tensin3-IDR-cBAK with either GFP-vinculin or GFP-talin1 in NIH3T3 cells reveals either protein is recruited to the tensin3 IDR at mitochondria. Scale bar indicates 5 µm. **(C)** Secondary structure prediction of the tensin3 IDR (aa490-1120). Red boxes indicate the H1 and H2 regions. **(D)** mCh-talinFL-cBAK was expressed in NIH3T3 cells together with a tensin3-IDR construct lacking either the H1 (GFP-tensin3-IDR∆H1) or H2 (GFP-tensin3-IDR∆H2) motif. Note the absence of co-localization between GFP-tensin3-IDR∆H1 and mCh-talinFL-cBAK, indicating that the H1 motif is responsible for this interaction. **(E)** A tensin3 construct lacking the H1 motif (GFP-tensin3-FL∆H1) co-expressed in NIH3T3 cells with either mCh-vinFL-cBAK or mCh-talinFL-cBAK is not recruited to mitochondria under either condition. Scale bars in B and C indicate 10 µm. Source data are available for this figure: SourceData FS3.

Following the BioID and mitochondrial targeting data plus our observations that FB formation is force-dependent (Fig. 1), we hypothesized a link between tensin and the mechanosensors talin and vinculin that connect integrins to the force-exerting actin cytoskeleton (Atherton et al., 2015). To identify binding regions, we generated a series of tensin3 truncations fused to GFP at the N-terminus (Fig. 3 A and Fig. S3 A). These included constructs lacking either the N- or C-terminal regions (“∆Nterm” and “∆Cterm”, respectively), and those consisting of the N- or C-terminal regions only (“Nterm” and “Cterm”). We first tested the recruitment of the GFP-tagged tensin truncations to mCh-vinFL-cBAK or mCh-talin-cBAK expressed in NIH3T3 fibroblasts. Only tensin3 constructs containing the central IDR (GFP-tensin3∆Nterm and GFP-tensin3-∆Cterm) were recruited (Fig. 3, B and C). This was surprising since the N-terminal PTEN-like region and C-terminal SH2-PTB regions had previously been thought to target tensins to FA (Liao and Lo, 2021). We, therefore, prepared constructs with the tensin3-IDR fused to cBAK or GFP and found that they associated with both talin and vinculin in mitochondrial targeting experiments (Fig. 3 D and Fig. S3 B). From these data, we concluded that talin and vinculin associate directly or indirectly with the tensin3-IDR.

### The IDR contributes to tensin3 localization to CMAs

The association of tensin3 with the FA proteins talin and vinculin through its IDR suggested that this region could contribute to the localization of tensin3 to CMAs. To test this possibility, we expressed the GFP-tensin deletion mutants in NIH3T3 cells and assessed their co-localization with endogenous vinculin. Both GFP-tensin3-∆Nterm and GFP-tensin3-∆Cterm are localized to CMAs (Fig. 3 E). Albeit fainter, both the GFP-tensin3-Cterm and GFP-tensin3-IDR constructs localized to vinculin positive adhesions, whereas GFP-tensin3-Nterm was not detected in CMAs (Fig. 3 E). These data lead to the conclusion that it is predominantly the central IDR and C-terminal SH2-PTB domains but not the N-terminus of tensin3 that are important for the recruitment of tensin3 to CMAs and for maximizing its efficient binding to adhesion components.

### A helical motif in the tensin3-IDR binds talin

**Figure 4.**
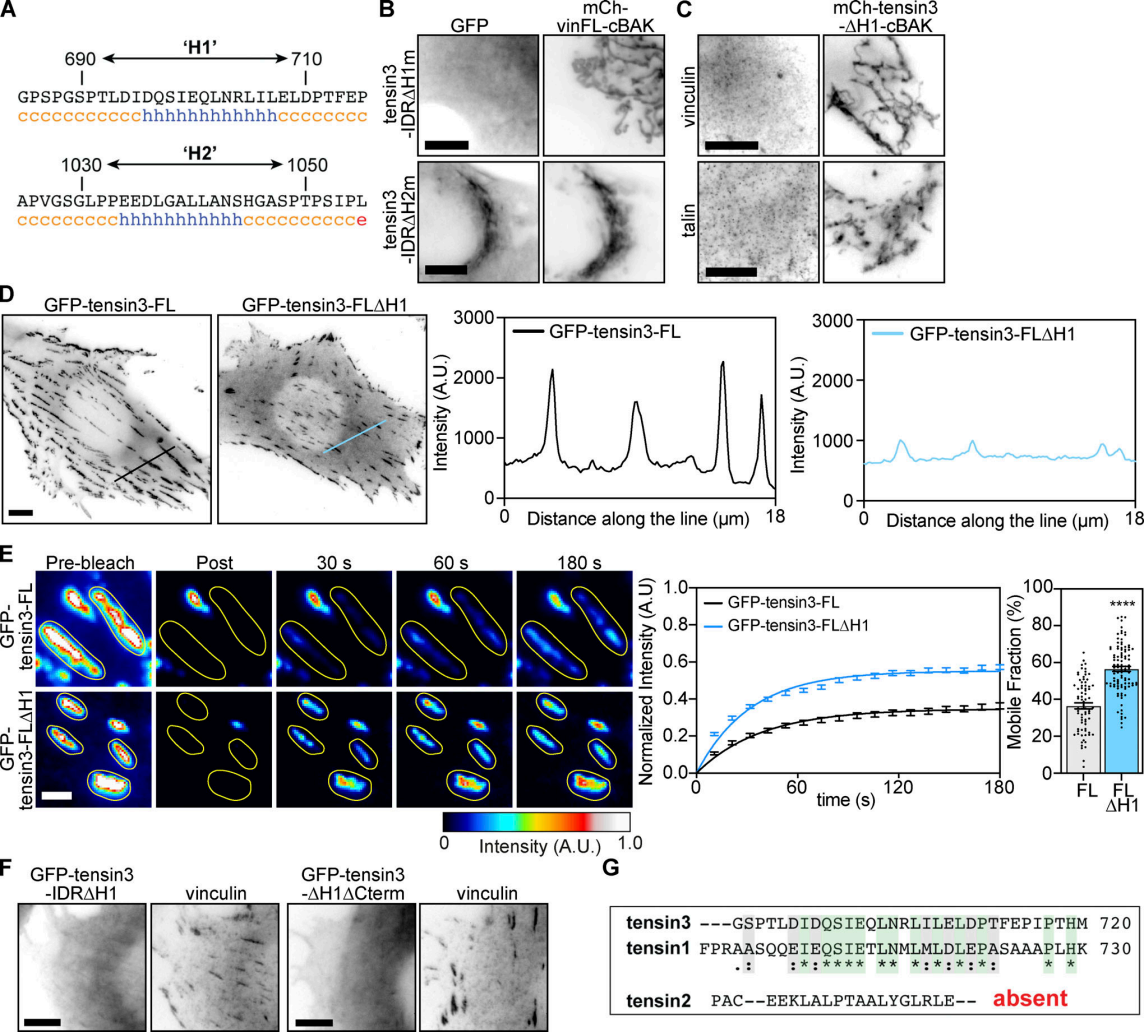
**A helical motif in the tensin3 IDR mediates the association with talin and vinculin. (A)** Secondary structure prediction of the tensin3 IDR reveals two stretches of amino acids predicted to form a helix, termed “H1” and “H2.” **(B)** Co-expression of mCh-vinFL-cBAK together with a tensin3-IDR construct lacking either H1 (GFP-tensin3-IDR∆H1) or H2 (GFP-tensin3-IDR∆H2) in NIH3T3 cells shows that the H1 motif is responsible for the interaction between the tensin3 IDR and vinculin. **(C)** Immunostaining of NIH3T3 cells expressing mCh-tensin3-∆H1-cBAK shows that the H1 motif is responsible for the recruitment of endogenous vinculin and talin to mitochondria. **(D)** Tensin3 lacking the H1 motif (GFP-tensin3-FL∆H1) expressed in NIH3T3 cells localization to cell-matrix adhesions. Line profiles show that this construct has a higher cytoplasmic fraction compared to GFP-tensin3-FL. **(E)** FRAP experiments in NIH3T3 cells show the H1 motif regulates the turnover of tensin3 within cell-matrix adhesion sites; mobile fraction 36.49% ± 1.62 and 56.51% ± 1.34. Error bars are SEM, *n* = 74 (GFP-tensin3-FL) or 99 (GFP-tensin3-FL∆H1) adhesions from 10 to 11 cells, pooled from two independent experiments; *** indicates P < 0.001 (*t* test). **(F)** Representative images of NIH3T3 cells expressing a tensin3 IDR construct lacking the H1 motif (GFP-tensin3-IDR∆H1) or a tensin3 construct lacking both the H1 motif and the C-terminal SH2-PTB domains (GFP-tensin3-∆H1∆Cterm). Note that neither of these constructs shows localization to vinculin-positive cell-matrix adhesion sites. **(G)** Amino acid sequence alignment of tensin1, tensin2 and tensin3 (using Clustal Omega) shows that there is some homology between the tensin3 H1 region and a corresponding stretch of amino acids in tensin1. No homology is observed in tensin2. Scale bars in B, C, D, and F indicate 10 µm.

To date, no structural domains or binding sites have been identified in the tensin–IDR that could account for binding to talin or any other FA protein. Examining the predicted secondary structure of the tensin3–IDR (Fig. S3 C) identified two short amino acid sequences containing a combination of hydrophobic and charged residues predicted to form helices (helices H1, residues 690–710 and H2, residues 1030–1050 of tensin3; Fig. 4 A). Mitochondrial targeting experiments using constructs consisting of the tensin3-IDR lacking either H1 or H2 (GFP-tensin3-IDR∆H1 or GFP-tensin3-IDR∆H2, respectively) revealed H1 to be responsible for the interaction of tensin3 with talin and vinculin (Fig. 4, B and C; and Fig. S3, D and E).

We next tested the contribution of H1 to tensin3 localization to CMAs. Widefield microscopy showed a reduced intensity of the GFP-tensin3-FL construct lacking H1 (GFP-tensin3-FL∆H1) in CMAs and higher levels in the cytoplasm when compared to the GFP–tensin3–FL (Fig. 4 D), suggesting reduced affinity to CMAs of the mutant tensin3. This was confirmed by the significantly increased mobility shown by an increase in the mobile fraction of GFP–tensin3–FL∆H1 in comparison to GFP–tensin3–FL in FRAP experiments (Fig. 4 E).

To gain further insight into the contribution of H1 for tensin3 localization, we prepared GFP-tensin IDR lacking the H1 motif (GFP-tensin3-IDR∆H1) as well as a GFP-tensin3-∆Cterm lacking H1 GFP-tensin3-∆H1∆Cterm. In contrast to the GFP-tensin3-IDR or GFP-tensin3-∆Cterm constructs that contained H1, deletion of H1 from either construct prevented their recruitment to CMAs (compare Fig. 4 F with Fig. 3 E). From these results, we conclude that both H1 and the C-terminus are the major sites that contribute to tensin recruitment to CMAs. Interestingly the H1 motif of tensin3 is conserved in the tensin1–IDR but has very low homology with the tensin2-IDR (Fig. 4 G), consistent with the similarity in tensin1 and tensin3 localization (Fig. S1). From these data, we conclude that the H1 motif likely mediates the association of tensin3 with talin and vinculin and that this motif contributes to tensin3 localization to CMAs.

### Tensin3 associates with vinculin indirectly through talin

**Figure 5.**
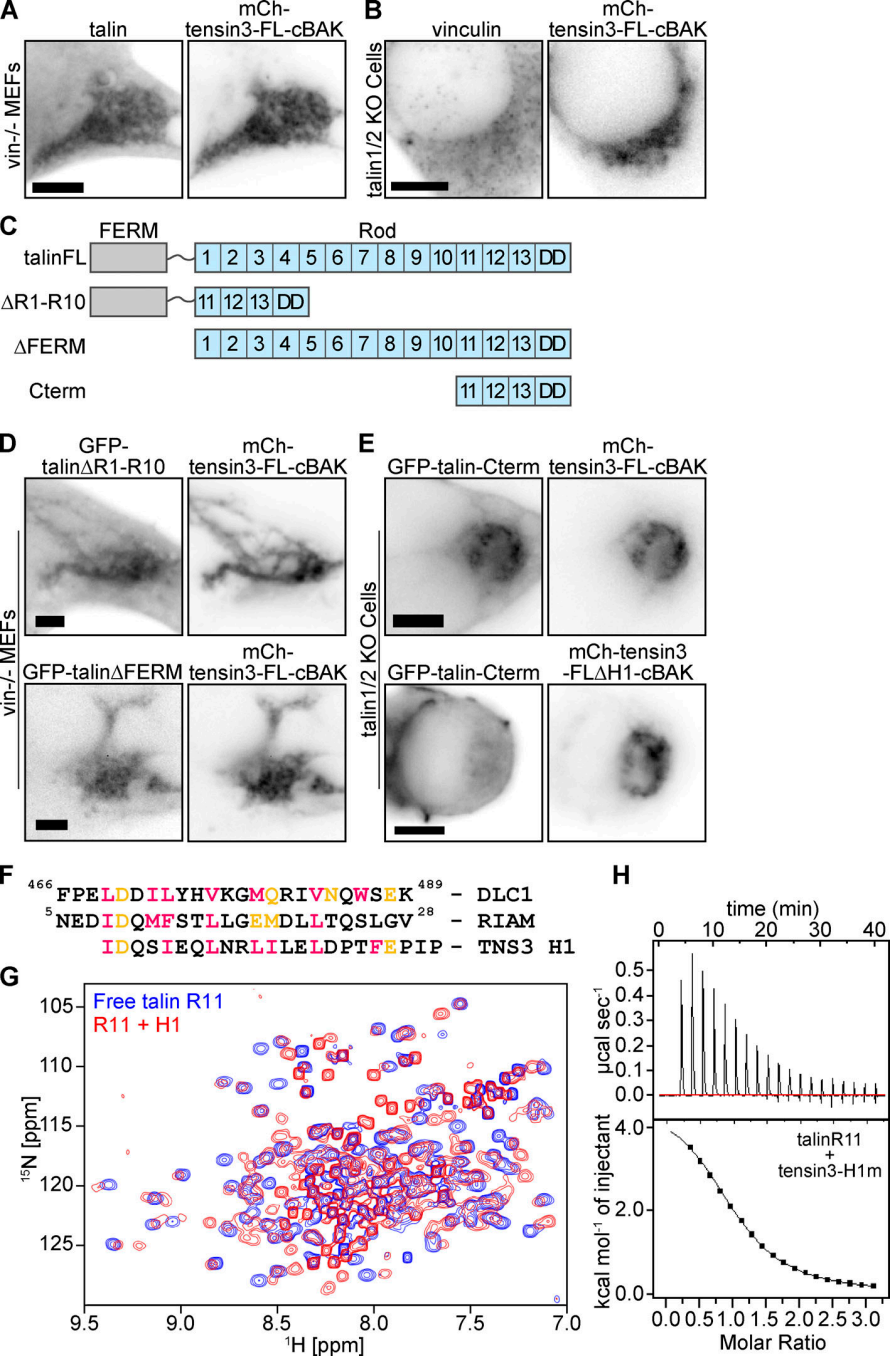
**Tensin3 H1 binds directly to the R11 domain of talin. (A and B)** Immunostaining of (A) endogenous talin in vinculinKO mouse embryonic fibroblasts (vin−/− MEFs) or (B) endogenous vinculin in talin1/2 KO cells expressing mCh-tensin3-FL-cBAK reveals that vinculin interacts with tensin3 indirectly through talin. **(C)** Schematic of talin constructs used. **(D)** Representative images of vin−/− MEFs cells expressing GFP-talin∆R1-R10 or GFP-talin∆FERM together with mCh-tensin3-FL-cBAK. **(E)** Representative images of talin1/2 KO cells expressing GFP-talin-Cterm with either mCh-tensin3-FL-cBAK or mCh-tensin3-FL∆H1-cBAK. **(F)** Sequence alignment of the talin binding sites in DLC1 and RIAM with the H1 sequence of TNS3. **(G)** ^1^H,^15^N-HSQC NMR spectra of 400 µM ^15^N-labelled isolated talin R11 in the absence (blue) and presence (red) of 1,600 µM of tensin H1 peptide. Note the decrease of signals in the complex due to the exchange broadening. **(H)** ITC measurements of tensin3 H1 interactions with talin R11. The experiments were conducted with 80 µM of R11 in cell and 1.2 mM of the peptide in the syringe. Scale bars in A, B, D, and E indicate 5 µm.

Our finding that both talin and vinculin require the same H1 region in the IDR for association (Fig. 4 C) raised the possibility that the association of tensin with one of them is direct and the other is indirect. To test this possibility, we performed mitochondrial targeting experiments in talin or vinculin knock-out cells. Co-localization of endogenous talin and mCh-tensin3-FL-cBAK expressed in vinculin knock-out mouse embryonic fibroblasts (MEFs) clearly shows that the talin/tensin3 interaction is vinculin independent (Fig. 5 A). In contrast, endogenous vinculin failed to associate with mCh-tensin3-FL-cBAK expressed in talin1 and 2 null cells (Fig. 5 B). This clearly indicates that tensin association with vinculin requires at least one of the talin isoforms.

**Figure S4.**
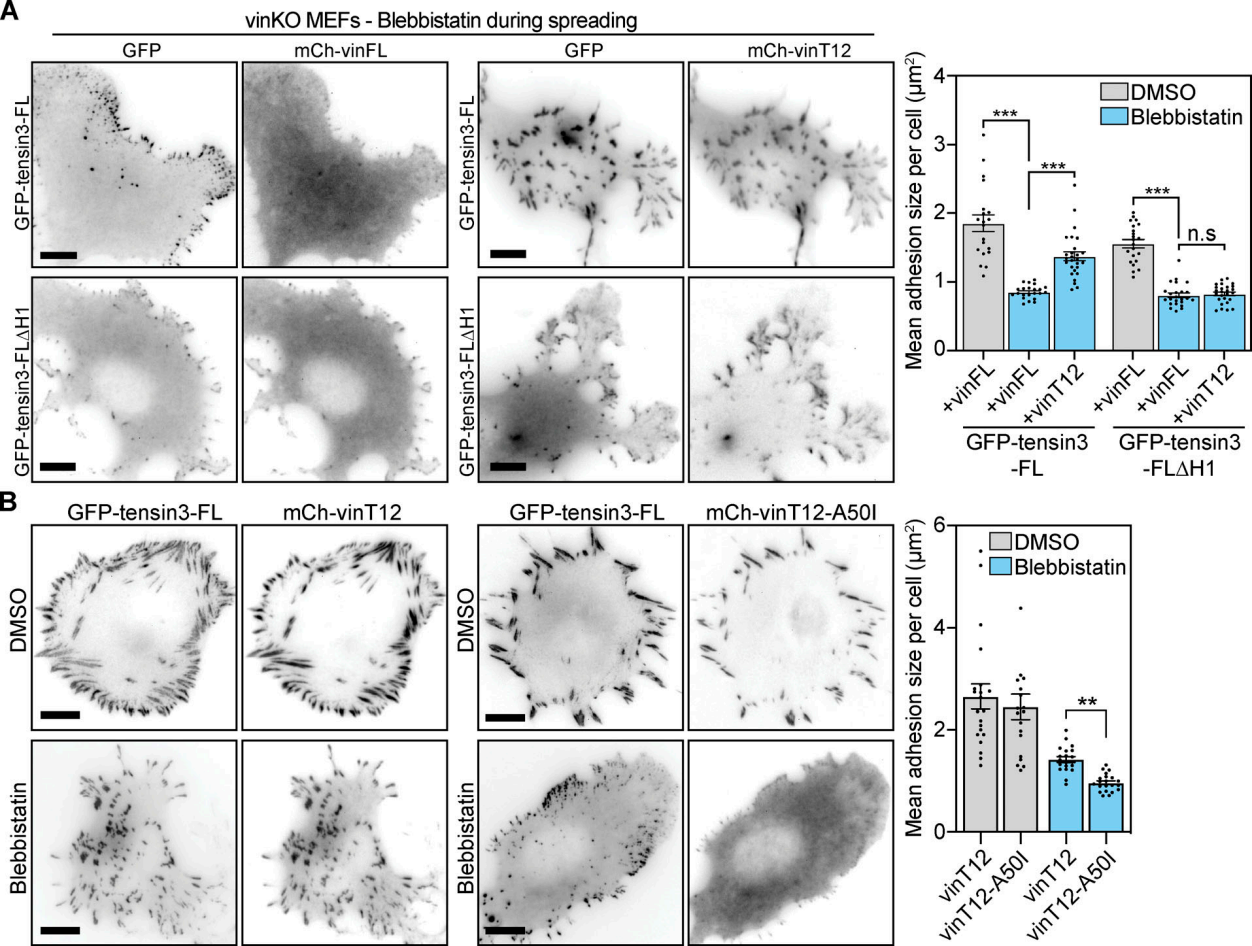
**Active vinculin can rescue tension-independent adhesion formation, but requires the vinculin-talin interaction. (A)** Representative images of vinculin-null cells (vinKO MEFs) co-expressing GFP-tensin3-FL or GFP-tensin3-FL∆H1 together with either mCh-vinFL or mCh-vinT12, treated in suspension with blebbistatin (50 µM) or an equivalent volume of DMSO, for 60 min. Cells were fixed after spreading on fibronectin-coated glass for 60 min. Quantification of mean adhesion size per cell using the GFP signal shows that co-expression of active vinculin (mCh-vinT12) can rescue formation of GFP-tensin3 positive adhesions under tension-free (blebbistatin-treated) conditions; this phenotype is not seen in cells expressing GFP-tensin3-FL∆H1. Error bars are SEM; *n* = 19–24 cells, *** indicates P < 0.001 (ANOVA), n.s. indicates not significant. **(B)** Representative images of vinculin-null cells (vinKO MEFs) co-expressing GFP-tensin3-FL together with either mCh-vinT12 or mCh-vinT12-A50I, treated in suspension with blebbistatin (50 µM) or an equivalent volume of DMSO, for 60 min. Cells were fixed after spreading on fibronectin-coated glass for 60 min. Quantification of mean adhesion size per cell using the GFP signal shows that inhibiting the vinculin-talin interaction (vinT12-A50I expression) blocks the formation of large GFP-tensin3-FL positive adhesions in tension-free conditions. Error bars are SEM; *n* = 19–21 cells, ** indicates P < 0.001 (Kruskal-Wallis test with Dunn’s multiple comparison test). Scale bars in A and B indicate 10 µm.

From these results, we hypothesized that vinculin could indirectly regulate tensin3 function via talin. To explore this, we expressed GFP-tensin3-FL together with either mCherry-tagged wildtype (mCh-vinFL) or constitutively active (mCh-vinT12; Cohen et al., 2005) vinculin in vinKO MEFs and measured adhesion formation after spreading on fibronectin in the presence of blebbistatin. Similar to our earlier observations in U2OS and TIF cells (Fig. 1), vinKO cells co-expressing GFP-tensin3-FL and mCh-vinFL formed few GFP-tensin3-FL-positive adhesions when spread on fibronectin in the absence of actomyosin-mediated tension (Fig. S4 A). In contrast, vinKO cells co-expressing mCh-vinT12 and GFP-tensin3-FL displayed large GFP-tensin3-FL positive adhesions. Importantly, this effect was not seen in cells co-expressing mCh-vinT12 and the GFP-tensin3-FL∆H1 construct (Fig. S4 A), which is not able to bind talin. This effect was not seen in cells expressing GFP-tensin3-FL together with an active vinculin construct containing a point mutation that disrupts talin binding (vinT12-A50I, Bakolitsa et al., 2004; Fig. S4 B), supporting the notion that vinculin regulates tensin3 indirectly via talin.

### Tensin H1 binds to the talin R11 rod domain

To identify the region in talin that binds the tensin H1 motif, we co-expressed a variety of talin deletion constructs (Fig. 5 C) in vinculin knock-out MEFs together with mCh-tensin3-FL-cBAK. Initially, we analyzed the recruitment of either a talin construct lacking the FERM domain (GFP-talin∆FERM) or a talin construct lacking the R1–R10 rod domains (GFP-talin∆R1-R10) to mCh-tensin3-FL-cBAK at mitochondria. Both talin constructs localized with mCh-tensin3-FL-cBAK at mitochondria (Fig. 5 D) implicating the C-terminal talin R11-R13 rod domains in the interaction. Indeed, experiments in talin1/2 KO cells confirmed that a talin R11–R13 construct (GFP-talin-Cterm) co-localized with mCh-tensin3-FL-cBAK at mitochondria (Fig. 5 E). Importantly, this GFP-talin-Cterm construct did not co-localise with mCh-tensin3-FL∆H1-cBAK (Fig. 5 E), confirming that it binds to the tensin IDR H1 motif.

Interestingly, the H1 motif shares similarities to the talin binding sites in RIAM and DLC1 (Fig. 5 F; Goult et al., 2013; Zacharchenko et al., 2016), suggesting that tensin may interact with the R11 domain of talin that incorporates a RIAM binding site (Goult et al., 2013). To test this hypothesis, we conducted NMR and ITC experiments using a synthetic peptide corresponding to tensin3 H1 (residues 692–718) and a recombinant talin R11 domain. The addition of the tensin3 H1 peptide to 15N-labeled talin R11 resulted in large changes in the ^1^H, ^15^N-HSQC spectrum that monitors signals of the backbone NH-groups of talin R11 (Fig. 5G). The majority of the signals changed position and decreased in intensity, with only a small number of signals remaining unchanged. This effect is characteristic of the interactions with a dissociation constant (Kd) in the µM range, as previously has been observed for the interactions of the talin rod domains with RIAM, DLC1, and paxillin peptides (Goult et al., 2013; Zacharchenko et al., 2016). The Kd was measured in the ITC experiments. When the talin R11 solution was titrated with a high concentration of the tensin3 H1 peptide, we observed concentration-dependent heat absorption (positive peaks) corresponding to the endothermic interaction (Fig. 5 H). Negligible heat change was observed at the end of the titration, indicating the binding site saturation. The heat dependence fitted well to a single-site binding model with a Kd of 17 µM and an enthalpy of 4.8 kcal/mol (Fig. 5 H). A similar endothermic interaction was detected for the binding of the RIAM peptides to the talin R2 and R3 domains (Goult et al., 2013). Together these data reveal that the H1 motif in the tensin3-IDR is a bona fide talin binding site (TBS) that interacts with the talin R11 domain with an affinity similar to other reported TBSs. From here on, we refer to the H1 motif as “TBS.”

### Tensin controls integrin activity in centrally located CMAs

Both talin and tensin bind and activate integrins (Calderwood et al., 2013; Georgiadou et al., 2017; McCleverty et al., 2007). However, whilst talin is absolutely critical for the formation of peripheral FAs and cell spreading through integrin activation (Atherton et al., 2015; Zhang et al., 2008), tensin seems less important for cell spreading, but is important for fibronectin fibrillogenesis associated with the translocation of α5β1 integrins toward the cell center (Pankov et al., 2000).

**Figure S5.**
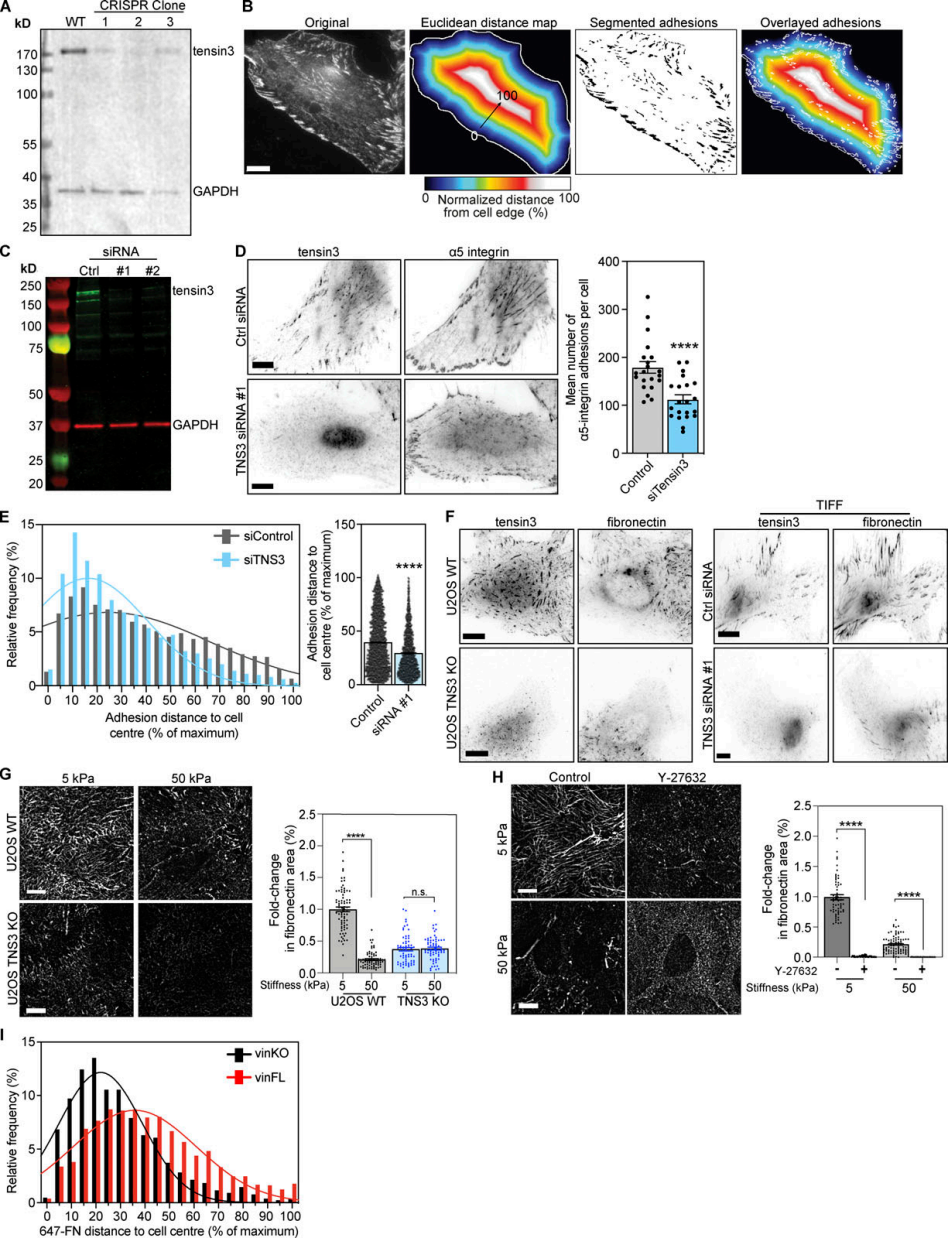
**Tensin3 is required for fibronectin fibrillogenesis. (A)** Generation of TNS3 KO cells using CRISPR; Western blot shows endogenous tensin3 levels in WT and 3 clonal populations generated following CRISPR. Clone #2 was a complete KO line (all cells were depleted of TNS3); clones #1 and #3 were a mixed population of KO and WT cells. **(B)** Analysis of (normalized) distance of adhesion structures from the cell periphery: a region of interest (ROI) was drawn around the edge of the cell to be analyzed. The ROI was filled with the Fill command, then the threshold function was used to select the pixels within the cell ROI, and converted to a binary image. The Distance Map function was used to create a Euclidean distance map (EDM), which was multiplied by the pixel size to convert to distance in µm. The resulting image was divided by the maximum distance value, then multiplied by 100 to convert to normalized distance between 0 and 100% (displayed here in a heat-map). Adhesion structures in the original image were segmented, and the resulting adhesion ROIs were applied to the normalized distance image. The mean intensity of each adhesion ROI was measured, which gives the normalized distance from the cell periphery, where 100 is the centre and 0 is the cell edge. Scale bar indicates 10 µm. **(C)** Western blot of endogenous tensin3 in TIFs after 2 rounds of transfection with either scrambled siRNA (Ctrl) or two different oligos targeting tensin3 separately (siRNA #1 or siRNA #2). Strongest knockdown was achieved with siRNA #1 alone, which was used for subsequent experiments. **(D)** Representative images of TIFs after siRNA-mediated knockdown of tensin3 as described above. Cells were cultured on fibronectin-coated glass overnight prior to fixation, then immunostained for α5 integrin. Quantification shows that tensin3 knockdown reduced the number of α5 integrin-positive adhesions. Errors bars are SEM; *n* = 14 (Control) or 16 (siRNA #1) cells; *** indicates P < 0.001 (*t* test). **(E)** Histograms and accompanying gaussian curve fit for the normalized distance (percent of maximum) of each a5-integrin positive adhesion structure from the cell edge to the cell centre; bar graphs show the mean and SEM of the normalized distance of all the adhesions; *n* = 3,913 (Control) and 2,450 (siTensin3) adhesions from 21 to 21 cells, respectively; **** indicates P < 0.0001, Mann-Whitney *t* test. Results are representative of two independent repeats. **(F)** Representative images of endogenous tensin3 co-localization with fibronectin in U2OS WT and TNS3 KO or TIFF control and TNS3 siRNA-treated cells. **(G)** Representative images (background subtracted) of fibronectin fibrils produced by U2OS WT or U2OS TNS3 KO cells spread overnight on fibronectin-coated soft (5 kPa) or stiff (50 kPa) polyacrylamide gels. Quantification of fibril formation (as performed for Fig. 7, A–C) normalized to WT cells on 5 kPa polyacrylamide gel shows that absence of tensin3 reduces significantly fibronectin fibril production on all substrates. Error bars are SEM, *n* = 63–77 squares from 30 to 35 images, pooled from three independent experiments; **** indiciates P < 0.0001 (Kruskal-Wallis ANOVA with Dunn’s multiple comparison test). **(H)** Representative (background subtracted) images of fibronectin fibrils produced by WT U2OS cells spread overnight on fibronectin-coated soft (5 kPa) or stiff (50 kPa) polyacrylamide gels in the presence of either DMSO (−) or Y-27632 (+). Quantification of fibril formation (as above) normalized to DMSO-treated cells on 5 kPa polyacrylamide gels shows that Y-27632 treatment dramatically reduces fibronectin fibril formation on all substrates. Error bars are SEM, *n* = 60–86 squares from 30 to 35 images, pooled from three independent experiments; **** indiciates P < 0.0001 (Kruskal-Wallis ANOVA with Dunn’s multiple comparison test). **(I)** Histograms and accompanying gaussian curve fit (related to Fig. 7 D) for the normalized distance (percent of maximum) of the distance of fibronectin (647-FN) fibers from the cell periphery formed by vinculinKO MEFs with or without expression of mCh-vinFL. Note that cells without vinculin have fewer centrally-located FN fibrils compared to cells expressing mCh-vinFL. Scale bars in E and F indicate 10 µm. Source data are available for this figure: SourceData FS5.

**Figure 6.**
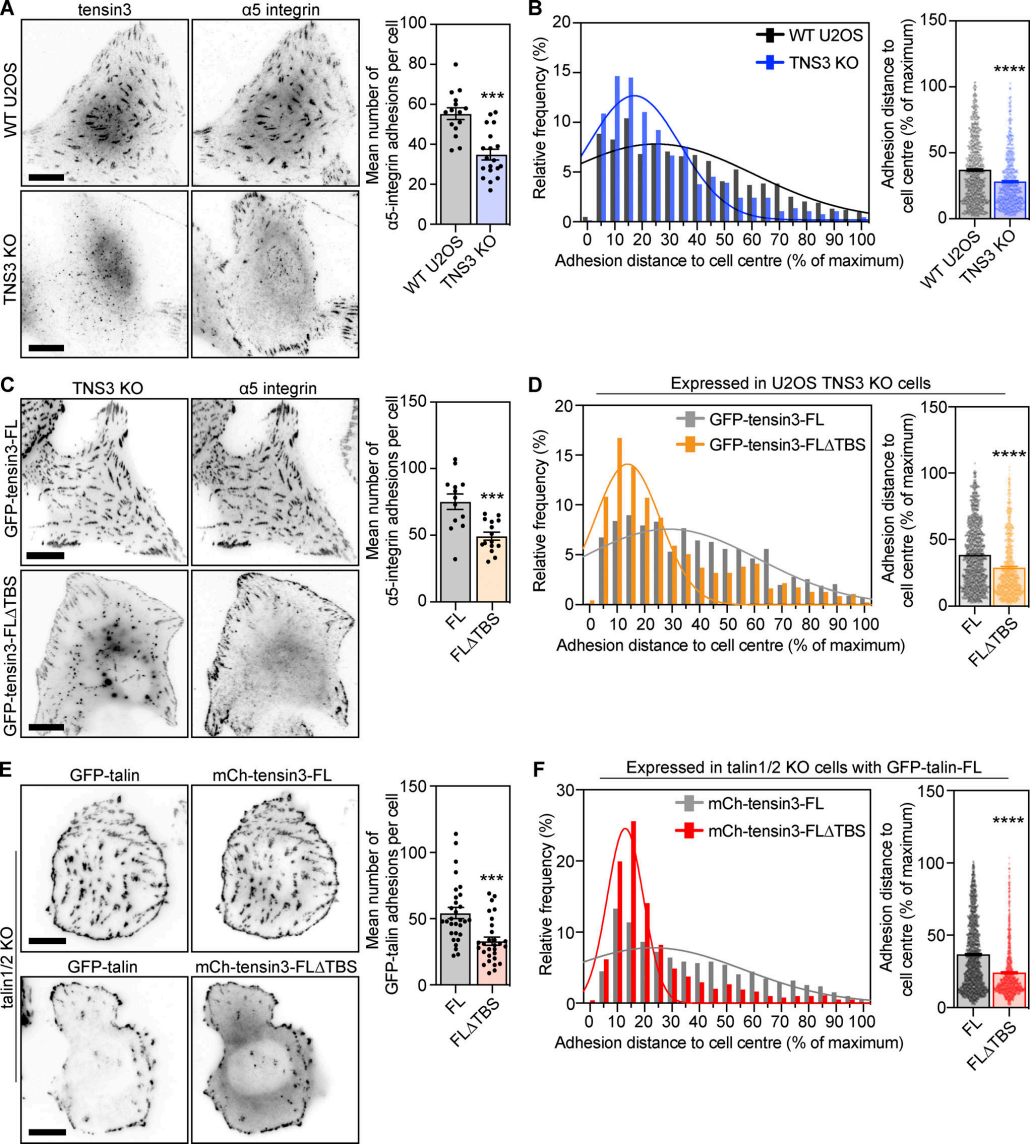
**The tensin3 talin binding site regulates α5 integrin localization. (A)** Representative images of wild-type (WT) or CRISPR-mediated tensin3 knock-down (TNS3 KO) U2OS cells spread overnight on fibronectin-coated glass before fixation and immunostaining for α5 integrin. Quantification shows that tensin3 KO cells (clone #2) have reduced α5 integrin-positive adhesions; error bars are SEM, *n* = 15 (WT) and 19 (KO) cells; *** indicates P < 0.001 (*t* test). **(B)** Histograms and accompanying gaussian curve fit for the normalized distance (percent of maximum) of each a5-integrin positive adhesion structure from the cell edge to the cell center; bar graphs show the mean and SEM of the normalized distance of all the adhesions; *n* = 836 (WT) and 662 (KO) adhesions from 15 to 19 cells, respectively; **** indicates P < 0.0001 (Mann-Whitney *t* test). Note that TNS3 KO cells have a significantly reduced number of centrally located adhesions. **(C and D)** Representative images of TNS3 KO cells expressing the indicated GFP-tagged tensin3 construct fixed and stained for α5 integrin. Number of adhesions (*n* = 13 [GFP-tensin3-FL] and 15 [GFP-tensin3-FL∆TBS] cells) and the distance from the cell periphery was quantified as above, revealing that expression of the GFP-tensin3-FL∆TBS construct is unable to rescue the formation of centrally located α5 integrin-positive adhesion structures. Note that while rescue with GFP-tensin3-FL resembles the WT U2OS phenotype, rescue with GFP-tensin3-FL∆TBS resembles the TNS3 KO phenotype. Error bars are SEM, *n* = 1,836 (GFP-tensin3-FL), 953 (GFP-tensin3-FL∆TBS) adhesions from 13 to 15 cells, respectively; *** indicates P < 0.001 (Mann-Whitney *t* test). **(E)** Representative TIRF images of talin1/2 KO cells co-expressing GFP-talin together with either mCh-tensin3-FL or mCh-tensin3-FL∆TBS. Quantification of the number of GFP-talin positive adhesions reveals fewer adhesion structures in cells expressing mCh-tensin3-FL∆TBS; *n* = 30 (mCh-tensin3-FL) and 29 (mCh-tensin3-FL∆TBS) cells; *** indicates P = 0.0002 (*t* test). **(F)** Histograms and accompanying gaussian curve fit for the normalized distance (percent of maximum) of each GFP-talin positive adhesion structure from the cell edge to the cell center; bar graphs show the mean and SEM of the normalized distance of all adhesions; *n* = 1,650 (WT) and 991 (KO) adhesions from 30 to 29 cells, respectively; **** indicates P < 0.0001 (Mann-Whitney *t* test). All results shown in A–F are representative of three independent repeats; scale bars indicate 10 µm.

To explore the role of tensin3 toward this process, we analyzed the distribution of α5β1 integrin-positive CMAs (SNAKA51 antibody staining) in WT and CRISPR-mediated tensin3 knock-out (TNS3 KO) U2OS cells (Fig. S5 A). Tensin3 knock-out cells had ∼40% fewer α5-integrin positive adhesions (Fig. 6 A). Measuring the distance of these adhesion structures from the cell periphery to the cell center (for details of adhesion distance analysis see Fig. S5 B) revealed that TNS3 KO cells had fewer α5-integrin positive adhesions located toward the cell center compared with WT U2OS cells, where the α5-integrin positive adhesions were distributed more evenly throughout the cells (Fig. 6 B). Similar results were seen when tensin3 was depleted using siRNA in TIFs (Fig. S5, C–E).

Next, we investigated the contribution of the tensin3–talin interaction towards α5β1 integrin localization by analyzing the distribution of α5-integrin positive adhesions in TNS3 KO cells expressing either GFP-tensin3-FL or GFP-tensin3-FL∆TBS. Cells expressing GFP-tensin3-FL∆TBS had ∼35% fewer α5-integrin positive adhesions compared to cells expressing GFP-tensin3-FL (Fig. 6 C). Similar to TNS3 KO cells, the α5-integrin positive adhesions of cells expressing GFP-tensin3-FL∆TBS were predominantly located toward the cell periphery rather than the cell center (Fig. 6 D). A similar phenotype of reduced CMAs at the cell center was observed in experiments using talinKO cells co-expressing GFP-talin with either mCh-tensin3-FL or mCh-tensin3-FL∆TBS, with a reduction in the number of GFP-talin positive adhesions (Fig. 6 E) and a striking absence of centrally-located CMAs in cells expressing mCh-tensin3-FL∆TBS compared to mCh-tensin3-FL (Fig. 6 F). It is thus the expression of tensin3 and the co-operation with talin that seem to regulate the abundance of FBs.

### The talin–tensin3 interaction regulates fibronectin fibrillogenesis

**Figure 7.**
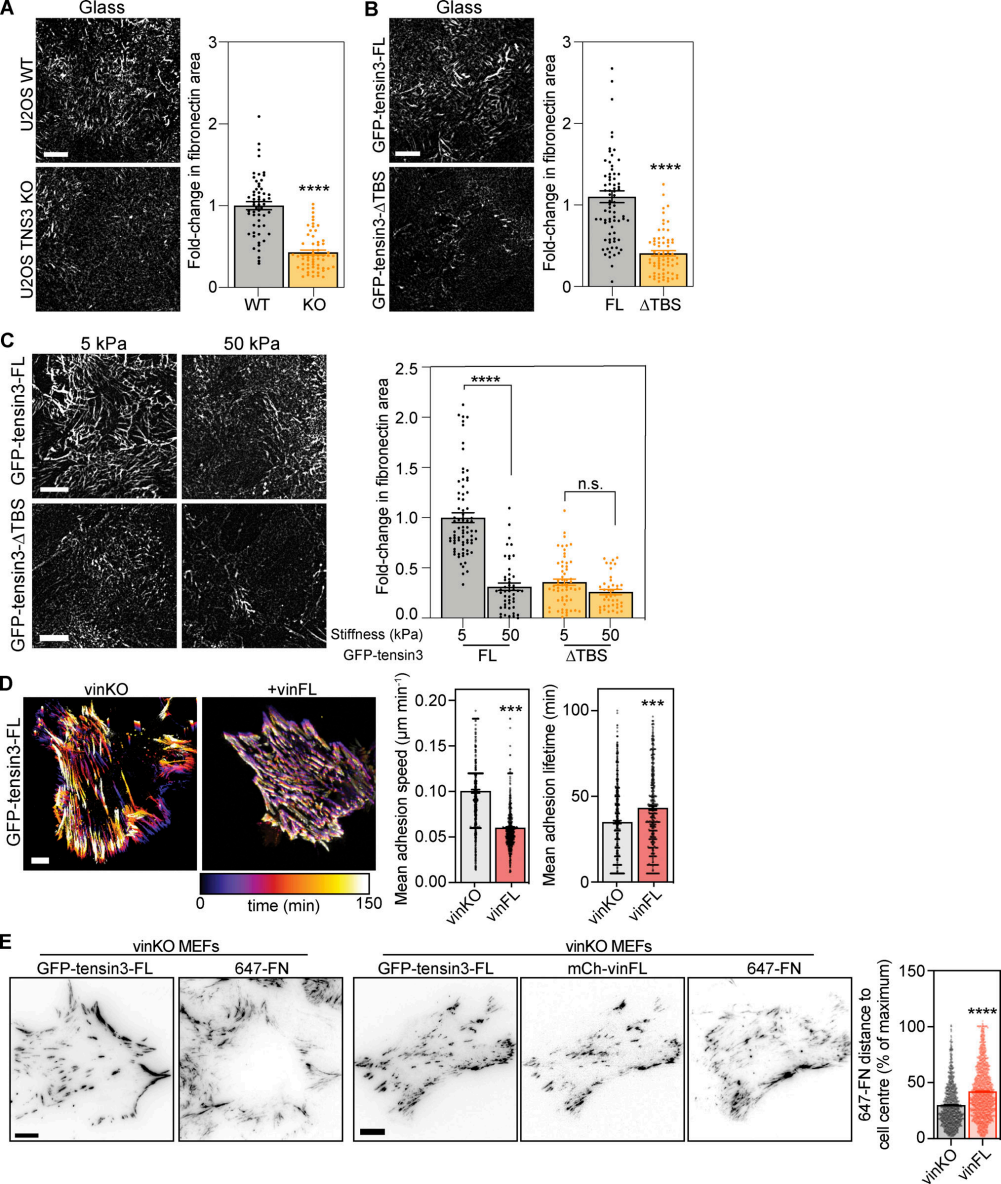
**Fibronectin fibrillogenesis is dependent on the tensin3–talin interaction. (A and B)** Representative (background subtracted) images of fibronectin fibrils (using an antibody against cell-derived fibronectin (Serini et al., 1998) produced by (A) WT or TNS3 KO U2OS cells or (B) TNS3 KO U2OS cells expressing GFP-tensin3-FL or GFP-tensin3-FL∆TBS, spread overnight on fibronectin-coated glass. Fibril formation was quantified in 70 × 70 µm squares applied to each image and is normalized to U2OS WT (A) or U2OS TNS3 KO expressing GFP-tensin3-FL (B) to calculate the fold-change in the area covered by fibronectin fibrils. Quantification shows 50% reduction in fibronectin fibrils produced by TNS3 KO (A) and GFP-tensin3-FL∆TBS (B) cells. Error bars are SEM, *n* = 57 (WT); 60 (KO); 76 (GFP-tensin3-FL); 69 (GFP-tensin3-FL∆TBS) squares from 30 to 35 images, pooled from three independent experiments; **** indiciates P < 0.0001 (Mann-Whitney *t* test). **(C)** Representative images (background subtracted) of fibronectin fibrils produced by TNS3 KO U2OS cells expressing GFP-tensin3-FL or GFP-tensin3-FL∆TBS, spread overnight on fibronectin-coated soft (5 kPa) or stiff (50 kPa) polyacrylamide gels (quantified as Fig. 7, A and B) normalized to GFP-tensin3-FL on 5 kPa polyacrylamide gels. Note the reduced fibronectin fibrils produced by GFP-tensin3-FL∆TBS expressing cells on both substrates. Error bars are SEM, *n* = 75 (GFP-tensin3-FL, 5 kPa); 48 (GFP-tensin3FL, 50 kPa); 58 (GFP-tensin3-FL∆TBS, 5 kPa); 41 (GFP-tensin3-FL∆TBS, 50 kPa) squares from 30 to 35 images, pooled from three independent experiments; **** indicates P < 0.0001 (Kruskal-Wallis ANOVA with Dunn’s multiple comparison test). **(D)** Live-cell imaging of vinKO MEFs expressing GFP-tensin3-FL with or without mCh-vinFL co-expression; temporal color maps of adhesion movement obtained from the GFP signal show that tensin3-positive adhesions in cells without vinculin are more dynamic. Images were acquired every 5 min for 2 h. Quantification of mean adhesion speed and lifetime from time-lapse movies of vinKO MEFs expressing GFP-tensin3-FL with or without mCh-vinFL co-expression. Error bars are SEM, *n* numbers are pooled from 6 (vinKO) or 10 (+vinFL) cells; *** indicates P < 0.001 (*t* test). **(E)** Representative images of vinKO MEFs expressing GFP-tensin3-FL with or without mCh-vinFL. Alexa Fluor 647-labeled fibronectin (647-FN) was added to the culture medium for 2 h before fixation. Quantification of the mean distance of 647-FN fibrils from the cell periphery shows that cells without vinculin have significantly fewer centrally located FN fibrils compared to cells expressing mCh-vinFL. Error bars are SEM; *n* = 1,318 (vinKO) or 1,875 (+vinFL) fibres from 24 (vinKO) or 22 (+vinFL) cells (see Fig. S5 I for accompanying histograms); **** indicates P < 0.0001 (*t* test). Data are representative of two independent experiments. Scale bars in A–D indicate 10 µm.

The translocation of fibronectin-bound α5-integrin from the cell periphery to the cell centre during FB maturation drives the formation of fibronectin fibrils (Clark et al., 2005; Pankov et al., 2000). Given that tensin3 is required for the maturation of centrally-located α5-integrin positive adhesions, we next tested the ability of cells lacking tensin3 to produce fibronectin fibrils. U2OS and TIFF cells were plated at a low confluence on fibronectin-coated glass and allowed to spread overnight. We detected a reduced number of fibronectin fibrils in U2OS TNS3 KO compared to WT, and TIFF siRNA control-treated cells compared to TNS3 siRNA-treated cells (Fig. S5 F). However, the amount of fibronectin produced by single cells was relatively low and thus difficult to quantify, so we then plated U2OS WT and TNS3 KO cells on fibronectin-coated glass bottom dishes again at high confluency (Fig. 7, A and B). U2OS TNS3 KO cells produced 65% lesser fibronectin fibrils compared to U2OS WT cells (Fig. 7 A), with a similar reduction observed between U2OS TNS3 KO cells expressing GFP-tensin3-FL∆TBS compared to GFP-tensin3-FL (Fig.7 B). Cells in vivo can encounter different stiffnesses, particularly during fibrotic diseases or in solid tumors. Therefore, we tested whether fibronectin fibrillogenesis (assessed by immunofluorescence using an antibody against cell-derived fibronectin [Serini et al., 1998] and quantifying the percent of the cell area covered by fibrils after overnight culture) were affected when U2OS cells were cultured on either soft (5 kPa) or stiff (50 kPa) polyacrylamide (PAA) gels. Interestingly, U2OS cells showed an ∼80%-fold increase in fibronectin fibrillogenesis on soft PAA gels compared to stiff PAA gels (Fig. S5 G). Compared to WT U2OS cells (soft PAA gel), TNS3 KO cells showed an ∼60%-fold decrease in fibronectin fibrillogenesis across all substrates, with no differences observed between soft and stiff PAA gels (Fig. S5 G). Rescue experiments in TNS3 KO cells showed that expression of GFP-tensin3-FL, but not GFP-tensin3-FL∆TBS, could restore fibronectin fibrillogenesis similar to levels observed for WT U2OS cells (Fig. 7 C). Intriguingly, GFP-tensin3-FL∆TBS cells also showed no differences between soft and stiff substrates (Fig. 7 C), similar to TNS3 KO cells.

To examine whether actomyosin-mediated forces are required for the observed fibrillogenesis, we cultured the cells overnight in presence of either blebbistatin or the Rho kinase inhibitor Y-27632. In both cases, fibronectin fibrillogenesis was completely blocked (Fig. S5 H for Y-27632; data not shown for blebbistatin). Together, these data indicate that softer substrates stimulate fibronectin fibrillogenesis, which is controlled by the talin–tensin interaction.

### Vinculin regulates tensin dynamics to control fibronectin fibrillogenesis

The above experiments demonstrate that the talin/tensin interaction, together with actomyosin-mediated forces, is key for efficient FB formation and fibronectin fibrillogenesis. Vinculin is a critical regulator of talin function and stability at CMAs (Atherton et al., 2015), and of actomyosin-mediated forces that act on FAs (Atherton et al., 2016). Therefore, we explored the contribution of vinculin to the regulation of tensin3-positive CMAs by analyzing the dynamics of GFP-tensin3-FL adhesions using live-cell imaging of vinKO MEFs expressing GFP-tensin3-FL with or without mCh-vinFL. Automated segmentation and tracking of the GFP-tensin3-FL signal revealed that the absence of vinculin increased the mean movement speed of GFP-tensin3-FL adhesions by ∼40% and reduced adhesion lifetime by ∼20% (Fig. 7 D and Video 1).

**Video 1.** **Related to** Fig. 7 D**.** Time-lapse recordings of GFP-tensin3 at CMAs in vinculinKO MEFs with or without vinFL co-expression. The video shows vinKO MEFs expressing GFP-tensin3-FL either without (left panel) or with (right panel) co-expression of mCh-vinFL (not shown) imaged using spinning disk confocal microscopy, with images collected every 5 min for 4 h. Note the increased stability of GFP-tensin3 positive adhesion structures in cell co-expressing mCh-vinFL, compared to cells lacking vinculin expression. Images are played back at 3 frames/s. Scale bar indicates 10 µm.

Since tensin and α5-integrin dynamics are thought to be involved in fibronectin fibrillogenesis (Pankov et al., 2000), we hypothesized that the reduced stability of tensin3-positive adhesions in vinculinKO MEFs could translate to impaired fibronectin fibrillogenesis in these cells. To assess this, we added fluorescently-labeled fibronectin (647-FN) to the culture medium of vinKO MEFs expressing GFP-tensin3 with or without mCh-vinFL co-expression and quantified fibril formation after 2 h. Analysis of the distribution of the distance of the 647-FN fibrils that had formed after 2 h (analyzed in the same manner as α5-integrin positive adhesions in Fig. 6) revealed that cells lacking vinculin had formed fewer 647-FN fibrils at the cell center compared to cells expressing mCh-vinFL (Fig. 7 E and Fig. S5 I). Taken together, our results lead to the conclusion that tensin3 associates with talin through the TBS in the tensin3 IDR, with vinculin functioning as an “enhancer” of the talin–tensin mediated adhesion maturation that is required for efficient fibronectin fibrillogenesis.

## Discussion

The cells’ ability to remodel their extracellular matrix is critical for tissue homeostasis and the aberrant production of ECM components is commonly observed across multiple cancer types (Cox, 2021) and in fibrosis (Herrera et al., 2018). CMAs are known to play a key role in matrix organization, and FBs are thought to have a major role in this process (Clark et al., 2005). The binding of α5β1 integrins to fibronectin dimers initiates fibronectin self-association, with actomyosin-mediated cellular forces driving further assembly into elongated, insoluble fibrils (Singh et al., 2010). Growth factor signaling, such as TGF-β, can drive this progression by increasing cellular contractility (Torr et al., 2015), and aberrant TGF-β signaling pathway is the hallmark of many fibrotic diseases (Pakshir et al., 2020). Fibrillar fibronectin assembly is accompanied by the maturation of FBs from FAs, a transformation associated with the enrichment of tensin3 alongside α5β1 integrin, both of which co-localize with fibronectin fibrils. Tensin(s) have been implicated in this process due to their enrichment at fibronectin-associated FBs (Clark et al., 2010; Katz et al., 2000; Sottile and Hocking, 2002; Zamir et al., 2000) and knock-down studies showing that their depletion impairs FN fibrillogenesis (Georgiadou et al., 2017). Additionally, tensin1 was found to be upregulated in TGF-β-induced myofibroblasts and idiopathic pulmonary fibrosis (IPF) lung samples (Bernau et al., 2017). Despite its well-documented presence at fibronectin-associated fibrillar adhesions, little is known about the mechanisms underpinning the recruitment of tensin to adhesions, or how this impacts fibronectin fibrillogenesis. Here, we shed light on the mechanisms regulating this phenomenon. We demonstrate that FB maturation and fibronectin fibrillogenesis is controlled by the interaction of the talin R11 rod domain with a helical TBS motif in the tensin3 IDR (Fig. 5). Abolishing this interaction by depleting cells of tensin3 or through deletion of the tensin3, TBS inhibits both α5β1 enrichment at FBs and fibronectin fibrillogenesis (Figs. 6 and 7). Furthermore, we show that vinculin associates with tensin3 indirectly through talin and acts to potentiate the tensin3-dependent FB maturation (Fig. 7) that drives fibronectin fibrillogenesis.

Our BioID data identified talin as a close tensin3 neighbor, providing initial clues about a possible interaction between tensin3 and the mechanosensory talin/vinculin complex. Whilst the proximity of tensin3 to both talin1 and 2 and KANK2 were novel findings, previous BioID experiments with FAK, vinculin, LPP, ILK, parvin (Chastney et al., 2020), paxillin, and kindlin2 (Dong et al., 2016) as a bait identified tensin3 as the prey. The pseudokinase PEAK1 was also recently identified as a direct interactor of tensin3 (Zuidema et al., 2022). How these multiple potential interactions with tensin3 are organized in time and space, how they contribute to the maturation process of FAs into FBs, and to what extent they collaborate to regulate tensin-associated functions (i.e., FN fibrillogenesis) remains speculative. However, one could imagine the following scenario(s) with the contribution of proteins that we have identified in the tensin “neighborhood” through BioID: talin, absolutely required for cell spreading (Atherton et al., 2015) engages at the cell periphery with integrins and the actomyosin force machinery and vinculin binding to talin reinforces this link. Tensin can be recruited through the binding to talin, ILK, or other proteins to focal adhesions, but since both talin and tensin bind to the same NPxY motif of integrin cytoplasmic domains, they may start to compete for this binding site during the retrograde movement of the adhesion toward the center of the cell. KANK2, by binding to talin1, was found to have a role in the gliding of KANK2-positive CMAs into α5β1 enriched adhesions and thus could promote the transition of FB into cell areas that experience lesser forces (Sun et al., 2016). KANK itself may contribute to the reduction of forces through its ability to diminish the talin–actomyosin linkage. However, since forces contribute to the maintenance of talin activity and integrin binding capability, talin may then lose its battle with tensins that are competing for the same NPxY motif of integrins. Mechanosensitive proteins such as talin and vinculin thus gradually leave and tensin becomes enriched in FBs. Another scenario could involve ILK binding to tensin (Qian et al., 2009). Stanchi and colleagues reported ILK as essential for adhesion site maturation to tensin-enriched FBs (Stanchi et al., 2009). A potential role for a direct interaction between tensins to ILK in the FA-to-FB maturation process is possible, but it is worth noting that ILK associates with all three tested tensins in our mitochondria recruitment assay (Fig. 2 D and Fig. S2 C), but only tensins 1 and 3 were found localizing to FBs (Clark et al., 2010 and our observation). Additional regulation may come from signaling pathways associated with tensin3 interactors such as FAK-Src signaling, and it is noteworthy that tensin fails to segregate into FBs in Src-null cells (Volberg et al., 2001), and inhibition of either FAK (Ilic et al., 2004) or Src signaling also impairs fibronectin fibril assembly (Wierzbicka-Patynowski and Schwarzbauer, 2002). Phosphorylation of the NPxY motif by Src has been proposed as a mechanism controlling the differential binding of talin or tensin to the cytoplasmic integrin tail, with phosphorylation acting to reduce the affinity for talin (but not tensin), leading to talin dissociation (McCleverty et al., 2007).

The mitochondrial targeting system confirmed the BioID outcomes and demonstrated that many of the identified neighbors form a complex with tensin3 independent of integrins which are absent from mitochondria (Fig. 2 and Fig. S2). The overall similarity in (i) tensin1 and 3 binding partners and (ii) their localization suggests that they have a similar function. However, the observation that tensin3 depletion leads to an almost complete loss of central adhesions demonstrates that tensin1 cannot compensate for the loss of tensin3. In contrast, our results for tensin2 were quite different: whilst all tested binding partners for tensin3 also associated with tensin1, only a few including paxillin and ILK bound to tensin2, suggesting that tensin2 has a quite distinct function.

Force-dependent maturation of peripheral FAs to central FBs was observed in early studies characterizing FBs as structures enriched in tensin, fibronectin, and α5β1 integrins (Pankov et al., 2000; Zamir et al., 2000). Our finding that tensin3 constructs lacking either the reported N-terminal actin-binding site or C-terminal integrin binding site localized to central adhesions similar to tensin3 wild-type (Fig. 3 E) suggest neither interaction drives the force-dependent enrichment of tensin3 at FBs. Rather, our data reveal that it is the newly identified tensin3–talin interaction (Fig. 5) that drives α5β1 positioning (Fig. 6) and fibronectin fibillogenesis (Fig. 7). Interestingly, the amino acid sequence of the TBS in tensin1 and 3 is also somewhat conserved in chicken tensin (unpublished observation), a region that overlaps with a region in the chicken tensin IDR that blocks FB formation, but was previously assigned to be an actin-binding region (Chuang et al., 1995; Pankov et al., 2000). Since none of our tested tensin constructs showed striking localization with actin stress fibers but rather connect to talin, we hypothesize that tensin experiences force indirectly through the link to talin, which when active associates with actomyosin (Atherton et al., 2015).

FBs were previously shown to be influenced by the stiffness of substrates they encounter. FBs increased in length up to levels of 7 kPa, but after this point, FBs remained stable or even decreased slightly (Barber-Perez et al., 2020). Our experiments showed unexpectedly high levels of FN fibrils on 5 kPa that decreased when cells were on stiff 50 kPa matrices (Fig. 7). It thus seems that the quantity of FN assembly does not necessarily reflect the number of FBs. Clearly, more experiments are needed to shed light on the mechanisms of this observation. One possibility is that fibronectin molecules coupled to a more pliable substratum require lower forces to unfold during fibrillogenesis. Such a model is similar to the one reported by Katz et al. (2000) who observed that the covalent binding of FN to surfaces blocks the transition of classical focal contacts to FN fibril-associated FBs. Such models could also influence a possible rigidity threshold for FN fibrillogenesis resembling that observed for the activation of the mechanosensory talin-vinculin complex (Elosegui-Artola et al., 2016). Alternatively, cells on a soft substratum may produce more fibronectin through alterations in gene transcription in an attempt to make a stiffer environment better resembling their native environment. Irrespective of substrate stiffness, it is clear that fibronectin fibrillogenesis is force sensitive since the blocking of actomyosin function abolishes this process (Fig. S5 H). The finding that vinculin-null fibroblasts had fewer centrally-located fibronectin fibrils (Fig. 7 E) and more dynamic tensin3-positive CMAs (Fig. 7 D) suggests vinculin contributes to the forces acting on the tensin3–talin complex, which govern α5β1 positioning and fibronectin fibrillogenesis as FAs transition to FBs. This is consistent with a previous study which shows that vinculin is required for force transmission and efficient ECM remodeling in 3D culture (Thievessen et al., 2015).

**Figure 8.**
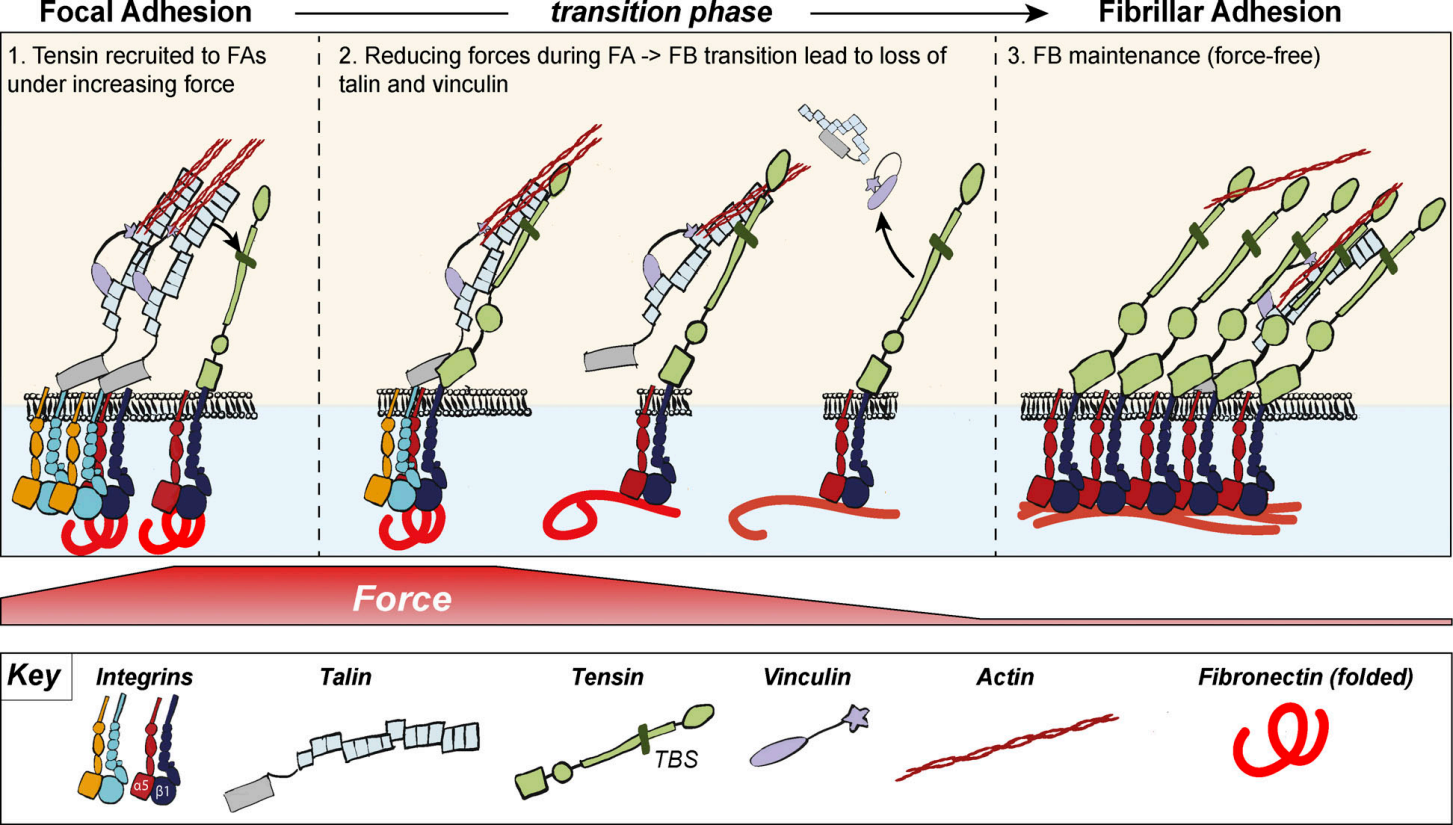
**Schematic model of tensin3 recruitment to CMAs and the maturation of focal adhesions to fibrillar adhesions.** At the cell periphery actomyosin links integrins to adhesion complexes through talin and vinculin to form FAs. Tensin joins this complex in FAs where it binds to talin and other adhesion complex proteins including integrins. Forces through actin binding to the mechanosensors talin/vinculin will cause the translocation of tensin to more central locations in the cell. This translocation depends on (i) tensin binding to talin via the talin binding site located in the tensin IDR, which engages R11 in the talin rod and (ii) the substrate rigidity, whereby fibronectin that associates with α5β1 integrins favours fibrilogenesis when cells encounter more pliable substrates. We speculate that binding of tensin3 to the cytoplasmic domain of α5β1 integrins competes with talin binding to integrins which then leads to a reduction of forces acting on the adhesion proteins, as observed in experiments using vinculin (LaCroix et al., 2018) or talin (Kumar et al., 2016) tension sensor constructs. The gradual reduction of forces across either of these mechanosensitive proteins may lead to their disassociation from centrally located adhesions, thus reducing the integrin-actin axis via the mechanosensors. In contrast, tensin3 remains present at FBs maintaining α5β1 integrins in an active, ligand-engaged state (Georgiadou et al., 2017) independently of tension. As such, there is a hand-over of talin-integrin to tensin3-integrin, resulting from the reduction of forces across the mechanosensitive talin molecule. The resulting stable integrin-tensin enriched structures remain attached to fibronectin fibrils and may represent a signalling hub that remains active to uphold tension-independent integrin-mediated cell-matrix communication processes required to maintain tissue function.

Improper ECM remodeling is increasingly becoming recognized as a hallmark of tissue diseases such as cancer and fibrosis. Our findings shed further light on the molecular mechanisms underpinning cellular control of ECM remodeling, and taken together, suggest a new model (Fig. 8), which explains how tensin3-enriched FBs form and how they are involved in ECM remodeling. Elucidating the mechanisms underpinning ECM homeostasis and how these go awry in diseases will hopefully lead to the identification of new therapeutic targets.

## Materials and methods

### Cell culture

NIH3T3 mouse fibroblasts and U2OS human osteosarcoma cell lines were obtained from the American Type Culture Collection (ATCC). The vinculin-/-MEFs originate from the Eileen A. Adamson laboratory (Xu et al., 1998). Telomerase immortalized fibroblasts (TIFs) were a gift of Pat Caswell (University of Manchester, Manchester, UK) and originate from the Brad Ozanne laboratory (Scott et al., 2004). All cells were cultured in Dulbecco’s modified Eagle medium (DMEM) supplemented with 10% FCS (Lonza), 1% l-glutamine (Sigma), and 1% non-essential amino acids (Sigma-Aldrich). Talin1&2 double null cells (Atherton et al., 2015) were cultured in DMEM:F12 (Lonza) supplemented with 10% FCS (Lonza), 1% l-glutamine (Sigma-Aldrich), 15 µM HEPES (Sigma-Aldrich), and 1% non-essential amino acids (Sigma-Aldrich). Transient transfections were performed using Lipofectamine 2000 and Lipofectamine Plus reagents (Invitrogen), as per the manufacturer’s instructions. siRNA sequences used for transient knockdown of human tensin3 are given in Table S2. For live-cell imaging and fixed cell imaging, cells were cultured on glass-bottom dishes (IBL) coated with bovine fibronectin (Sigma-Aldrich) diluted in PBS at a final concentration of 10 µg ml^−1^.

### Antibodies and reagents

Samples were fixed in 4% paraformaldehyde (PFA), which was pre-warmed to 37°C, for 15 min before being washed thrice with PBS. For immunofluorescence, samples were permeabilized at room temperature with Triton X-100 (0.5%) for 5 min before being washed thrice. Primary and secondary antibodies used are given in Table S2. Blebbistatin (Tocris Bioscience) was diluted in DMSO (Sigma-Aldrich) and used at a final concentration of 50 µM. Y27632 (Tocris Bioscience) was diluted in dH_2_O and used at a final concentration of 50 µM. Mitotracker Deep Red FM (Thermo Fisher Scientific) was dissolved in DMSO to a concentration of 1 mM. Before use, the stock was diluted in pre-warmed medium at a final concentration of 200 nM before being added directly to cells 30 min prior to fixation. Site-directed mutagenesis was performed using the QuikChange Lightning site-directed mutagenesis kit (Agilent) according to the manufacturer’s instructions.

### Protein extraction and Western blot

Cells from a six-well plate were lysed in 150 μl lysis buffer (25 mM HEPES pH 7.3, 150 mM NaCl, 5 mM MgCl_2_, 1 mM EDTA, 20 mM β-glycerophosphate, 5% glycerol, 0.5% Triton X-100, protease inhibitor). Then, 20 μg of protein was loaded on 7.5% SDS-PAGE gel. The gel was transferred to a nitrocellulose membrane, which was blocked in 5% milk. The membrane was probed for anti-tensin1, anti-tensin2, anti-tensin3, anti-GAPDH, or anti-GFP. Primary antibody signal was detected using HRP-conjugated secondary antibodies imaged with an Azure c400 imaging system (Azure Biosystems) or using IRDye–conjugated secondary antibodies (LI-COR Biosciences) imaged with an Odyssey imaging system (LI-COR Biosciences).

### Plasmid preparation

pEGFP-Tensin1, 2, and 3 plasmids were generated as described in Clark et al. (2010). The BioID vector pCDH-TagBFP-T2A-myc-BirA* (generous donation from Andrew Gilmore’s lab (The University of Manchester, Manchester, UK) was linearized (2 μg) by cutting with Xhol and BamH1. Gibson Assembly was used to design the primers to replace Xhol to BamHI region of the vector backbone with tensin3 (fragment 1: 2228 bp, forward primer: 5′-TGG​ATG​GGC​GGA​GAA​ATC​TCC​CTG​AGA​AGC​TCG​GGT​GGG​TCC​GGC​GGT​GGC​TCT​GGC​ATG​GAG​GAG​GGC​CAT​GG-3′, reverse primer: 5′-CCG​AGG​GCG​TTC​ATG​TGG​GTA​GGG​ATG​GGC​TC-3′; fragment 2: 2246 bp, forward primer: 5′-CAC​CTT​CGA​GCC​CAT​CCC​TAC​CCA​CAT​GAA​CG-3′, reverse primer: 5′-TCG​ACT​CAG​CGG​TTT​AAA​CTT​A AGC​TTG​GTA​CCG​AGC​TCG​TCA​GAC​CTT​CTT​TGG​TGA​ACC​AAT​CAT​GAC​C-3′). The two tensin fragments were amplified individually by PCR using KOD Xtreme Hot Start DNA Polymerase (Sigma-Aldrich) and were run on 1% agarose gel along with the linearized vector. Bands of the correct size were cut out and purified using the Isolate II PCR and gel kit (Bioline).

A total of 50 ng of vector and 150 ng of each fragment were mixed with 3 μl of HiFi DNA Master Mix (NEB) and incubated at 50°C for 1 h to form the new pCDH-TagBFP-T2A-myc-BirA*-Tensin3 construct. Bacterial transformation was then carried out using C2987 cells with 250 μl SOC medium. The DNA was extracted using QIAprep Spin Miniprep Kit (Qiagen). All enzymes were supplied from New England Biolabs.

### CRISPR RNP KO of tensin

The gRNA complex was prepared by mixing 1 μl of tensin 3 crRNA (100 μM; 5′-AGU​CCG​CUC​CCG​CUC​AUA​G-3′; Sigma-Aldrich) and 1 μl of trRNA (100 μM; 1072533; IDT) with 98 μl of nuclease-free water to prepare a complex of 100 μM final concentration. The complex was heated for 5 min at 95°C to assemble the complex and was then left to cool on the bench to room temperature.

The transfection complex was prepared by mixing gRNA complex with 1 μΜ Cas9 nuclease V3 (1081058; IDT) at a ratio of 1.3:1 in Opti-MEM and incubated for 5 min at room temperature. The gRNA:Cas9 complex was mixed with lipofectamine 2000, used per the manufacturer’s instructions, and incubated for 20 min at room temperature. Reverse transfection was performed by adding the transfection complex to freshly plated cells (RNP final concentration, 10 nM). Limited dilution was performed after 48 h of incubation with the transfection complex to collect individual cells which were expanded and tested for tensin3 expression by immunofluorescence and Western blot.

To assess CRISPR efficiency, DNA was extracted from the cells and sequenced. The sequences were uploaded on ICE to assess how many cells were missing the targeted sequence.

### Generation of stable cell lines

U2OS cells were co-transfected with pCDH-TagBFP-T2A-myc-BirA* or pCDH-TagBFP-T2A-myc-BirA*-tensin3 along with a puromycin vector at a 5:1 ratio. After 24 h of transfection, cells were trypsinized and diluted at a ratio of 1:5 before replating. Stable cells were selected with 1.5 μg ml^−1^ puromycin (1 mg ml^−1^ stock; Invitrogen) 24 h after replating. Upon colony formation, individual blue clones were isolated using colony rings (Sigma-Aldrich) and screened by cell imaging and Western blot before expanding. Stable cells were then supplemented with 0.5 μg ml^−1^ puromycin until the cell line was fully established and then expanded to 10-cm dishes.

Established stable cells were habituated in either heavy (H) or control light (L) isotopic labeled DMEM for SILAC (Thermo Fisher Scientific) for 14 d (six passages) supplemented with 10% dialyzed FBS (Thermo Fisher Scientific), 1% Pen/Strep, 143 mg ml^−1^ lysine, and 83 mg ml^−1^ arginine (K8R10 “Heavy” or K0R0 “Light”). In the last passage step, 2–4 dishes were kept for each of the control and tensin3 cell lines, depending on how strong the expression was. In our case, four 10-cm dishes of BFP-myc-BirA*-tensin3 and two 10-cm dishes of BFP-myc-BirA* (due to stronger expression) were used. We performed the experiment in both directions with BFP-myc-BirA*-tensin3 adapted in “heavy” labeled medium combined with BFP-myc-BirA* adapted in “light” labelled medium and vice versa.

### BioID and NeutrAvidin affinity capture

Labeled cells with heavy or light SILAC medium were in situ labeled with D-biotin (100 μM, B20656; Invitrogen) overnight (16 h). The next day the cells were washed three times with phosphate-buffered saline (PBS; 5 min, room temperature) to remove the excess biotin. Cell were lysed in 500 μl of lysis buffer (25 mM Tris [pH 8.0], 100 mM KCl, 5% glycerol, 0.5% Triton X-100, 0.5% deoxycholate (DOC), 1 mM EDTA, and protease inhibitor cocktail; Roche) per 10 cm dish. The cells were disrupted using a p1000 tip. The lysates were then centrifuged at maximum speed (21,130 *g*) for 1 min to remove nuclei, and the supernatant was transferred to a fresh 1.5-ml tube. The lysates were made up to 0.2% SDS and sonicated for 20 s at 15% power using a tapered tip before they were centrifuged (13,200 rpm) for 10 min at 4°C. At this stage, the heavy-labeled samples were mixed with light-labeled samples and added to 100 μl Neutravidin beads slurry, which has been previously equilibrated with lysis buffer and incubated with rolling overnight at 4°C. The next day the samples were centrifuged at 4,000 rpm for 2 min to collect the beads. The supernatant was removed and the beads were washed with 500 μl lysis buffer + 0.2% SDS for 15 min at 4°C. The beads were then washed twice in wash buffer (25 mM Tris [pH 8.0], 50 mM NaCl, 1% SDS, and protease inhibitor cocktail) for 10 min at room temperature and 4°C, respectively. Proteins bound to Neutravidin beads were eluted in 80 μl of 1 × lithium dodecyl sulfate (LDS) sample buffer (Novex NuPAGE) with 1 mM biotin by heating at 95°C for 5 min. This was removed and replaced by 40 μl of distilled water which was heated with the beads at 95°C for another 5 min to rinse the beads. The 40 μl were then added to 80 μl of the previous elute and they were reduced to 40 μl under vacuum to concentrate the sample. Purified proteins (35 μl) were run on a 10% SDS-PAGE gel (1 mm) and stained with colloidal Coomassie Brilliant Blue (CBB, Novex; Invitrogen). Those with a weight >28 kD (Streptavidin dimer) were excised, processed for trypsin digestion using in-gel digestion procedures (Shevchenko et al., 2006), and the interaction candidates were identified using mass spectrometry. Protein enrichment was calculated as a heavy/light peptide ratio and used for analysis as described in Dong et al. (2016).

### Protein identification by SILAC mass spectrometry

Tryptic peptides were analyzed using an EASY-nLC 1,000 coupled to a Q Exactive Hybrid Quadrupole-Orbitrap (Thermo Fisher Scientific). The peptides were resolved and separated on a 50 cm analytical EASY-Spray column equipped with pre-column over a 120-min gradient ranging from 8 to 38% of 0.1% formic acid in 95% acetonitrile/water at a flowrate of 200 nl/min. Survey full scan MS spectra (m/z 310–2000) were acquired with a resolution of 70 k, an AGC target of 3 × 0^6^, and a maximum injection time of 10 ms. The top 20 most intense peptide ions in each survey scan were sequentially isolated to an ACG target value of 5e4 with a resolution of 17,500 and fragmented using a normalized collision energy of 25. A dynamic exclusion of 10 s and isolation width of 2 m/z were applied. Data analysis was performed with MaxQuant (Cox and Mann, 2008) version 1.6.0.1 using default settings. Database searches of MS data used Uniprot human fasta (2020 Jan release, 96817 proteins) with tryptic specificity allowing a maximum of two missed cleavages, two labeled amino acids, and an initial mass tolerance of 4.5 ppm for precursor ions and 0.5 Da for fragment ions. Cysteine carbamidomethylation was searched as a fixed modification, and N-acetylation and oxidized methionine were searched as variable modifications. Maximum false discovery rates were set to 0.01 for both protein and peptide. Proteins were considered identified when supported by at least one unique peptide with a minimum length of seven amino acids. Proteins with a ratio count <4 were excluded from the filtered data (Table S1), while mitochondrial proteins, histones, and biotin-related proteins were excluded from the potential binding partner list (Fig. 2 B). The mass spectrometry proteomics data have been deposited to the ProteomeXchange Consortium via the PRIDE (Perez-Riverol et al., 2019) partner repository with the dataset identifier PXD026343.

### Soft/stiff polyacrylamide gels and drug treatments

#### Polyacrylamide gels

Glass-bottom dishes (20 mm glass, #1.5; cellvis) were cleaned with 0.1 M of NaOH for 5 min, followed by aminosalinyzation by (3-Aminopropyl)triethoxysilane (APES; 440140; Sigma-Aldrich) treatment for 4 min. Then 4 ml PBS was added to dilute APES before it was removed from the dishes by excessive washing with water. Finally, the dishes were incubated with 0.5% glutaraldehyde diluted in PBS for 30 min at room temperature (RT). Dishes were washed with water and placed in 70% ethanol overnight. The next day the dishes were removed from ethanol and air-dried under a sterile tissue-culture hood. Meanwhile, 18 mm round coverslips (MARI0117580’ Marienfeld) were incubated with 50 μg/ml fibronectin (F1141; Sigma-Aldrich) diluted in PBS for 1 h at RT. Soft and stiff gels were prepared with 30% acrylamide solution (EC-890, Protogel, 30%, 37.5:1 ratio acrylamide/bis-acrylamide solution, National Diagnostics) diluted in PBS (ratio was adjusted for soft and stiff), 10% ammonium persulfate (A3678; Sigma-Aldrich), and 0.01% (v/v) tetramethylethylenediamine (TEMED; T9281; Sigma-Aldrich). To prepare a thin gel layer, 10 μl of each gel mixture was placed in the middle of the glass-bottom dish and the FN-coated coverslip was placed on the top (FN-coated side on the gel). The dishes were incubated for 30 min at 37°C to facilitate the transfer of FN onto the gel surface. Finally, the gels were covered with PBS for another 30 min and then the coverslip was removed. After three washes with PBS, cells were plated on the gels and cultured overnight at 37°C.

### Microscopy

#### Live-cell imaging

Images of transfected vinKO MEFs (prepared as described above) were acquired on a spinning disk confocal microscope (CSU-X1; Yokagowa) supplied by Intelligent Imaging Innovations, Inc. (3i) equipped with a motorized XYZ stage (ASI) maintained at 37°C, using a 100x/1.45 Plan Apo oil objective (Zeiss) and an Evolve EMCCD camera (Photometrics). One hour before imaging, the medium was changed to pre-warmed Ham’s F-12 medium supplemented with 25 mM HEPES buffer, 1% FCS, 1% penicillin/streptomycin, and 1% L-glutamine, with 5 mM Mn^2+^ added as appropriate. The 488 and 561 nm lasers were controlled using an AOTF through the laserstack (Intelligent Imaging Innovations [3I]).

#### Fluorescence recovery after photobleaching (FRAP)

Transfected NIH3T3 fibroblasts were incubated overnight at 37°C. The cells were placed in the microscope chamber at 37°C for 1 h before imaging to ensure they were in equilibrium. Images were collected on a Leica Infinity TIRF microscope using a 100x/1.47 HC PL Apo Corr TIRF Oil objective with a 488-nm diode TIRF laser (with 100% laser power and a penetration depth of 120 nm), an ORCA Flash V4 CMOS camera (Hamamatsu) with 400 ms exposure time, a camera gain of 2, and a Leica QwF-S-T filter cube. Leica LAS X software was used to bleach five to seven adhesions per cell (using regions of interest manually drawn around adhesions); three pre-bleach images were acquired, followed by one image every 10 s for 5 min post-bleach. Movies were analyzed using FIJI-ImageJ to extract intensity over time data. Intensity values were normalized to the first post-bleach value; normalized data were fit to a one-phase association model Y=Y0 + (Plateau-Y0)*(1-exp(-K*x)) using GraphPad Prism. Coefficients of the curve fit were extracted and transformed to generate mobile fraction (Mf) and half-time of recovery (t1/2 FRAP) as described previously (Carisey et al., 2011).

#### Fixed-sample imaging

Images of fixed samples in PBS were acquired at room temperature using a Zeiss AxioObserver Z1 wide-field microscope equipped with a 100×/1.4-NA oil objective and an Axiocam MRm camera, controlled by Zeiss Axiovision software. Samples were illuminated using a mercury bulb; specific band-pass filter sets were used to prevent bleed-through from one channel to the next (for GFP, 38HE [Zeiss] and for mCherry, 43HE [Zeiss]). Images of fixed U2OS samples in PBS were acquired at room temperature using a Leica DM6000 B wide-field microscope equipped with a 63x/1.4 oil objective and a photometrics coolsnap EZ camera, controlled by metamorph software. Samples were illuminated using the external light source for fluorescence excitation; Leica EL6000 (mercury metal halide lamp); specific band-pass filter sets were used to prevent bleed-through from one channel to the next (for GFP, L5 [Leica]; for mCherry, TX2 [Leica]; for DAPI, A4 [Leica]).

### Analysis of cell-matrix adhesions and fibronectin fibrils

Cell-matrix adhesion size, number, and percent area were quantified using FIJI-ImageJ (Schindelin et al., 2012) as described previously (Atherton et al., 2015), by subtracting background signal using a rolling ball algorithm, followed by thresholding to select adhesion structures and the Analyze Particles function of FIJI-ImageJ to quantify adhesions. Fibronectin staining images were quantified similarly, by quantifying the amount of fibronectin in 70 × 70 µm square boxes applied to cells within each image.

The distance of adhesions or fibronectin fibrils from the cell edge was quantified by using a region of interest drawn around the cell periphery, which was used to create a Euclidean distance map (EDM) using the Distance Map function in ImageJ. The EDM was then normalized between 0 and 100% by dividing the EDM by the maximum distance value and then multiplying by 100. Adhesions were segmented and thresholded as above and used to create masks that were applied to the EDM. The mean intensity in each adhesion mask applied to the EDM gives the distance from the cell edge (see Fig. S5 B).

Adhesion dynamics were quantified from live-cell imaging experiments (using the spinning disk confocal set-up described above) by using the TrackMate plugin for FIJI-ImageJ (Tinevez et al., 2017) for automated particle segmentation and tracking.

### In vitro peptide binding assays

#### Peptide and protein preparation

Recombinant wild-type mouse talin1 fragment R11 (residues 1,974–2,140) was previously cloned into pET151/D-TOPO expression vector (Gingras et al., 2009). Protein was produced in BL21 STAR (DE3) cultured in Luria-Bertani or 2xM9 minimal medium containing 1 g/l ^15^N-labeled NH_4_Cl and purified using nickel-affinity chromatography followed by ion exchange. Synthetic peptide corresponding to TNS3 H1 fragment (residues 692–712) was purchased from ChinaPeptides (Shanghai). The peptide was dissolved in MilliQ water at a concentration of 10 mM and pH was adjusted to neutral by adding NaOH. Required amount of the stock peptide solution was added to buffer for ITC or NMR sample of talin for the titration experiments.’

#### NMR spectroscopy

NMR spectra were collected on Bruker Neo 800 MHz spectrometers equipped with TCI CryoProbe. Experiments were performed at 298 K in 20 mM sodium phosphate (pH 6.5) and 50 mM NaCl with 5% (v/v) ^2^H_2_O. Spectra were processed with TopSpin (Bruker).

#### Isothermal titration calorimetry (ITC)

ITC experiments were performed using an ITC-200 (Microcal). ITC titrations were performed in 20 mM sodium phosphate (pH 6.5) and 50 mM NaCl and 0.5 mM TCEP (tris-carboxyethyl-phosphine) at 25°C. Data were integrated and fitted to a single-site-binding equation using Origin 7 software with an integrated ITC module (Microcal).

### Graphs and statistical analysis

All graphs were made using Prism 8 (GraphPad). Statistical analyses were performed using Prism 8 (GraphPad). Where appropriate, statistical significance between two individual groups was tested using a (two-tailed) *t* test. To test for significance between two or more groups, a one-way ANOVA was used with a Holm-Sidak’s multiple comparison test with a single pooled variance. Data distribution was tested for normality using a D’Agostino & Pearson omnibus K2 test; a P value >0.05 was used to determine normality.

### Online supplemental material

Fig. S1: Related to Fig. 1. Validation of specificity of tensin1, 2, or 3 antibodies; expression and localization of the different tensin family members in U2OS and TIF cells; blebbistatin experiments performed in TIF cells. Fig. S2: Related to Fig. 2. Localization of BirA-tensin3 to cell-matrix adhesions and biotinylation (detected by streptavidin) of these structures; representative images of mitochondrial recruitment experiments using mCh-tensin1, 2, or 3-cBAK. Fig. S3: Related to Figs. 3 and 4. Expression of GFP-tagged tensin3 constructs; tensin3-IDR secondary structure prediction and identification of helices H1 and H2 and their contribution to talin/vinculin binding of tensin3. Fig. S4: Related to Fig. 5. Vinculin regulates tensin3 indirectly via talin. Fig. S5: Related to Figs. 6 and 7. KO (CRISPR) and KD (siRNA) of tensin3 in U2OS and TIFs, respectively, leads to reduced centrally-located α5-integrin positive adhesions and impairs fibronectin fibrillogenesis. Video 1: Related to Fig. 7 D. Video of GFP-tensin3 dynamics in vinculinKO MEFs with or without mCh-vinculin co-expression. Table S1: Related to Fig. 2, A and B. Proteomic identification of tensin3 proximal proteins identified by BioID. Table S2: List of antibodies and siRNA sequences used.

## Supplementary Material

Table S1 Proteomic identification of tensin3 proximal proteins.

Table S2 List of antibodies and siRNA sequences used in this study.

SourceData FS1 is the source file for Fig. S1.

SourceData FS3 is the source file for Fig. S3.

SourceData FS5 is the source file for Fig. S5.
