# Supplementary material for: Tensin3 interaction with talin drives the formation of fibronectin-associated fibrillar adhesions
Source: J Cell Biol. 2022 Sep 8;221(10):e202107022. doi: 10.1083/jcb.202107022 (PMC9462884; doi:10.1083/jcb.202107022)
Supplement: SourceData FS1 — is the source file for Fig. S1. [file JCB_202107022_SourceDataFS1.pdf]

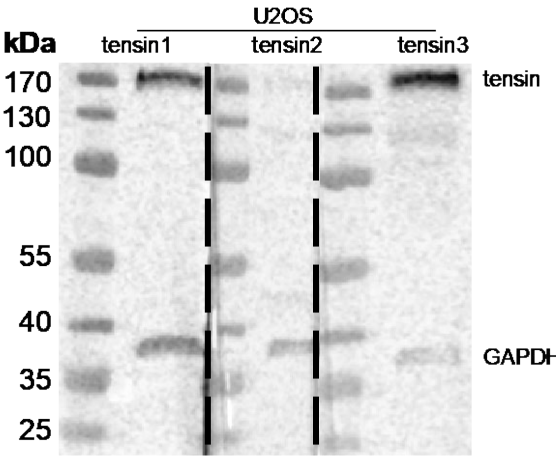

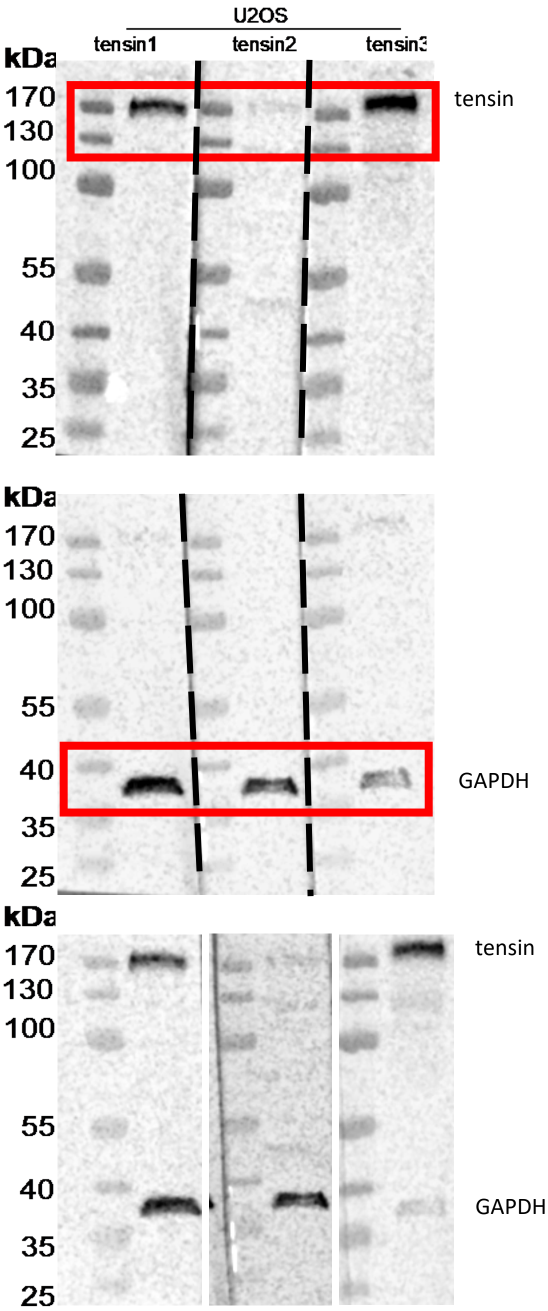

Uncropped merged images for Supp. Fig. 1
(Chemiluminescence + Colorimetric)
Red boxes indicate cropped blot shown in Supp. Fig. 1
Dotted line indicates where the membrane was cropped prior to antibody incubation
