# Supplementary figures and images for: Tensin3 interaction with talin drives the formation of fibronectin-associated fibrillar adhesions
Source: J Cell Biol. 2022 Sep 8;221(10):e202107022. doi: 10.1083/jcb.202107022 (PMC9462884; doi:10.1083/jcb.202107022)

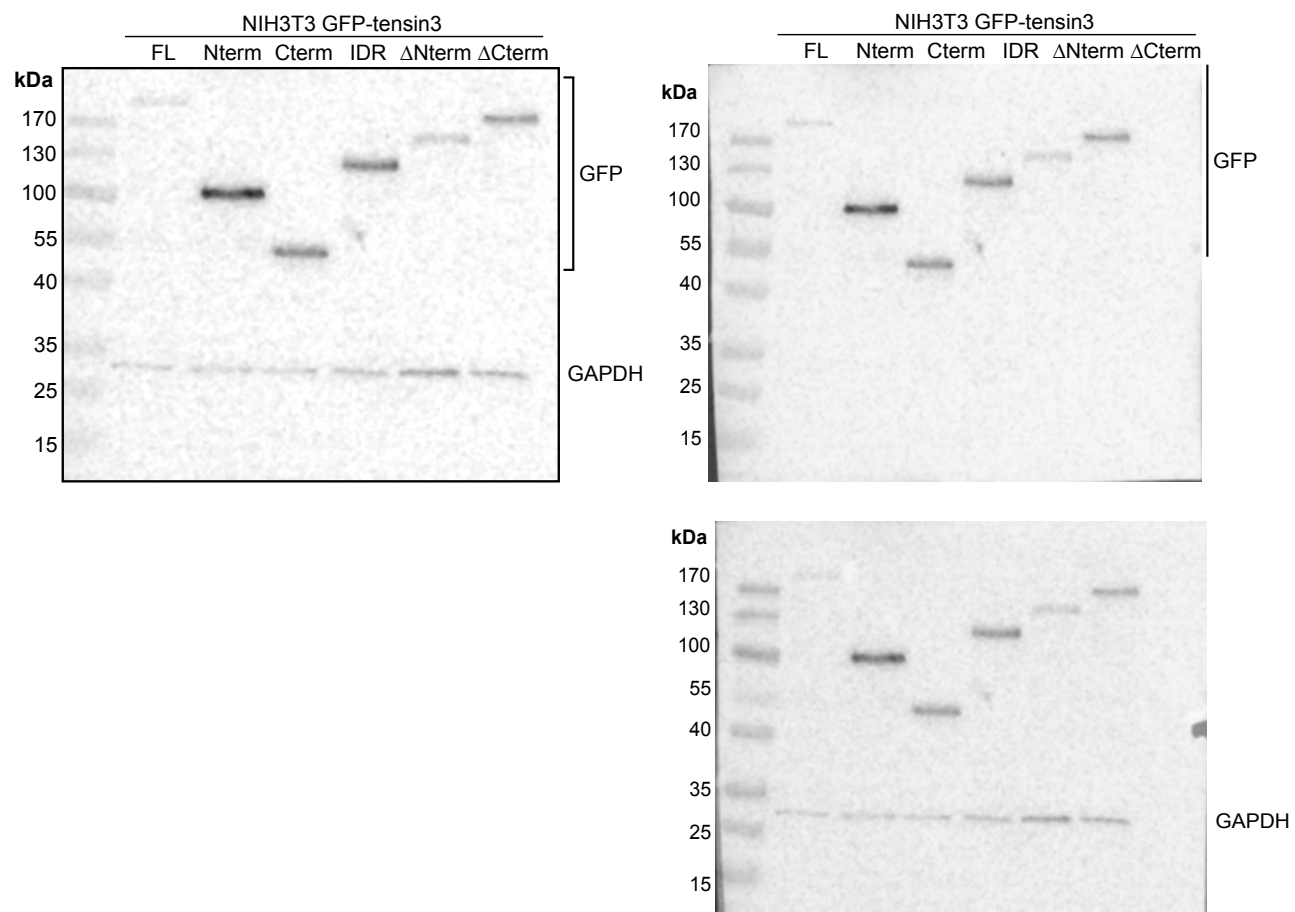

Uncropped merged images for Supp. Fig. 3  
(Chemiluminescence + Colorimetric)

Supplement: SourceData FS3 — is the source file for Fig. S3. [file JCB_202107022_SourceDataFS3.pdf]
