# Supplementary material for: Tensin3 interaction with talin drives the formation of fibronectin-associated fibrillar adhesions
Source: J Cell Biol. 2022 Sep 8;221(10):e202107022. doi: 10.1083/jcb.202107022 (PMC9462884; doi:10.1083/jcb.202107022)
Supplement: SourceData FS5 — is the source file for Fig. S5. [file JCB_202107022_SourceDataFS5.pdf]

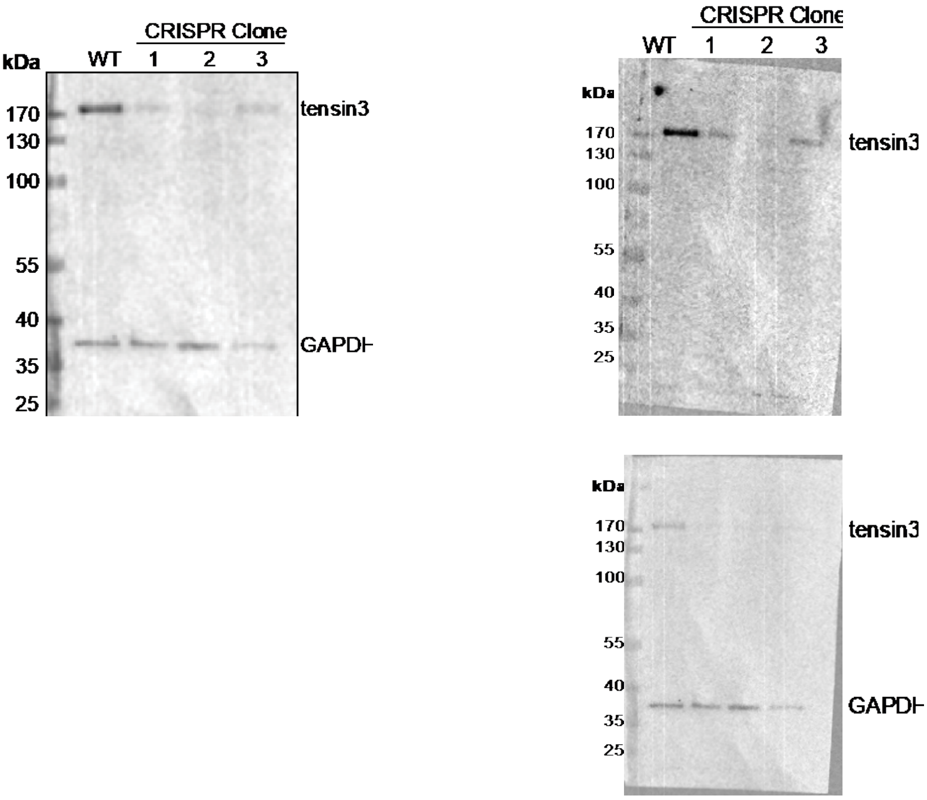

Uncropped merged images for Supp. Fig. 5A
(Chemiluminescence + Colorimetric)

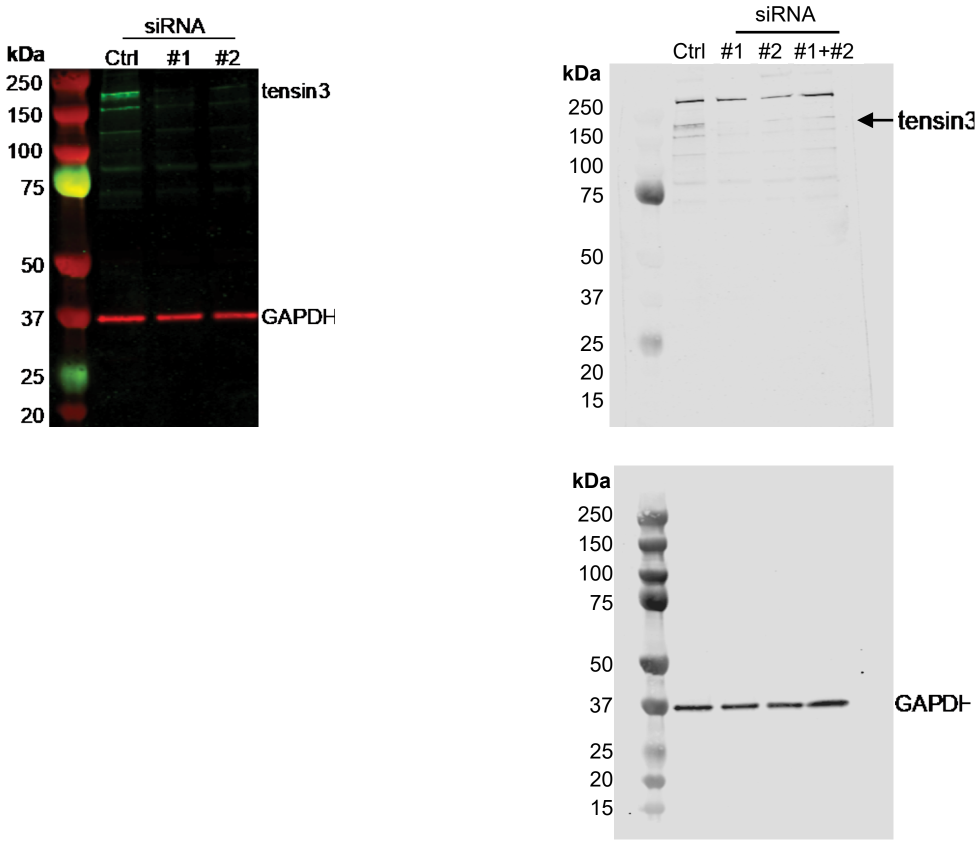

Uncropped merged images for Supp. Fig. 5C
(Colorimetric: red, GAPDH, 37kDa; green, tensin3, 170kDa)
